# Light entrainment of the SCN circadian clock and implications for personalized alterations of corticosterone rhythms in shift work and jet lag

**DOI:** 10.1038/s41598-021-97019-7

**Published:** 2021-09-09

**Authors:** Yannuo Li, Ioannis P. Androulakis

**Affiliations:** 1 Chemical & Biochemical Engineering Department, Rutgers, Piscataway, USA; 2 Biomedical Engineering Department, Rutgers, Piscataway, USA; 3 Departmnet of Surgery, Rutgers-RWJMS, Piscataway, USA

**Keywords:** Computational biology and bioinformatics, Systems biology

## Abstract

The suprachiasmatic nucleus (SCN) functions as the central pacemaker aligning physiological and behavioral oscillations to day/night (activity/inactivity) transitions. The light signal entrains the molecular clock of the photo-sensitive ventrolateral (VL) core of the SCN which in turn entrains the dorsomedial (DM) shell via the neurotransmitter vasoactive intestinal polypeptide (VIP). The shell converts the VIP rhythmic signals to circadian oscillations of arginine vasopressin (AVP), which eventually act as a neurotransmitter signal entraining the hypothalamic–pituitary–adrenal (HPA) axis, leading to robust circadian secretion of glucocorticoids. In this work, we discuss a semi-mechanistic mathematical model that reflects the essential hierarchical structure of the photic signal transduction from the SCN to the HPA axis. By incorporating the interactions across the core, the shell, and the HPA axis, we investigate how these coupled systems synchronize leading to robust circadian oscillations. Our model predicts the existence of personalized synchronization strategies that enable the maintenance of homeostatic rhythms while allowing for differential responses to transient and permanent light schedule changes. We simulated different behavioral situations leading to perturbed rhythmicity, performed a detailed computational analysis of the dynamic response of the system under varying light schedules, and determined that (1) significant interindividual diversity and flexibility characterize adaptation to varying light schedules; (2) an individual’s tolerances to jet lag and alternating shift work are positively correlated, while the tolerances to jet lag and transient shift work are negatively correlated, which indicates trade-offs in an individual’s ability to maintain physiological rhythmicity; (3) weak light sensitivity leads to the reduction of circadian flexibility, implying that light therapy can be a potential approach to address shift work and jet lag related disorders. Finally, we developed a map of the impact of the synchronization within the SCN and between the SCN and the HPA axis as it relates to the emergence of circadian flexibility.

## Introduction

Most organisms have evolved an endogenous circadian timing system to anticipate periodic variations in their environment. In mammals, the central pacemaker is located in the suprachiasmatic nucleus (SCN), which regulates physiological rhythms to be coordinated with the daily changes in the environment in a hierarchical manner. A set of mutually regulated genes and proteins within the SCN neurons form a self-sustained regulatory network that oscillates with a circadian period ($\approx$ $24 \; \text{h}$). This transcriptional-translational oscillator consists of positive and negative feedback loops. In particular, the transcription factor heterodimer BMAL1/CLOCK activates transcription of the *period* and *cryptochrome* genes (*Per1*, *Per2*, *Cry1*, and *Cry2*) by binding to the E-box promoter region^1^. Subsequently, the cytoplasmic proteins PER and CRY accumulate and form the PER/CRY heterodimer complex which translocates to the nucleus and downregulates its synthesis by inhibiting the activity of BMAL1/CLOCK^1,2^. In a positive feedback loop, *Bmal1* transcription is regulated by PER and CRY through the downregulation of *Rev-erb* $\alpha$ transcription^3,4^.

The SCN can be divided into two distinct sub-regions based on anatomical differences and neurochemical content: a ventrolateral core and a dorsomedial shell region surrounding the core. Neurons of the core contain several neurotransmitters: vasoactive intestinal polypeptide (VIP), calretinin, neurotensin (NT), and gastrin-releasing peptide (GRP), with VIP the most prevalent neurotransmitter. VIP is released rhythmically from the core and induces *Per1* and *Per2* expression in neurons for both the core and the shell by acting at the receptor VPAC2^5,6^, accomplishing the synchronizing function of the SCN core. The shell is characterized by neurons containing arginine vasopressin (AVP) colocalized with gamma aminobutyric acid (GABA)^7^. Directly below the SCN, the optic chiasm is located which projects the photic input to the SCN. In the core, the non-oscillatory calbindin (CalB)-containing cells are retinorecipient, thereby process light-input directly from the optic chiasm, subsequently conveying photic information to the oscillatory cells in the core. The core in turn transduces the entraining information to the shell through “master synchronizer” neurotransmitter VIP^8,9^. The core projects densely to the shell, while the shell projects sparsely back to the core, enabling photic information transduction indirectly to the shell through the core^10–12^.

Entrained by the central pacemaker (SCN), clock genes are also expressed rhythmically in peripheral tissues, primarily mediated via the rhythmic regulation of glucocorticoids (GCs) secreted by the hypothalamic–pituitary–adrenal (HPA) axis^13^. The hypothalamic paraventricular nucleus (PVN) releases a corticotrophin-releasing hormone (CRH) which activates the release of adrenocorticotropic hormone (ACTH) from the pituitary gland and in turn induces the secretion of GCs (cortisol in humans, corticosterone in rodents) from the adrenal glands^14^. Subsequently, GCs suppress upstream regulatory pathways in the PVN and the pituitary gland mediated through glucocorticoid receptors, thus forming a negative feedback loop resulting in the robust oscillation of GCs. This process is synchronized to photic cues (i.e., entrained by light) by the SCN-derived neurotransmitters. AVP exerts negative control over basal plasma corticosterone (CORT) concentrations by inhibiting the secretion of CRH macroscopically, resulting in a precise systemic circadian pattern with GCs peaking at the beginning of the active phase (day for humans, night for rodents)^15,16^. Eventually, the rhythmically produced HPA hormones play a central role in the response to environmental variations, enabling the host to actively re-establish homeostasis^17^. When the HPA axis becomes distorted upon exposure to irregular photic signals, allostatic load accumulation results in GC phase shifts and amplitude dampening which may potentially result in numerous pathologies^18,19^.

The importance of understanding the dynamics of photic entrainment of circadian rhythms motivates the need for characterizing the endogenous pathways, interactions between the SCN and the HPA axis, and allostatic state of the system in adverse environments. Considering the difficulty in measuring fluctuations of the physiological state of the host, mathematical modeling approaches can be particularly helpful in generating the experimentally verifiable hypotheses and deciphering the underlying dynamics to predict clinical outcomes and intervention strategies.

In this paper, we proposed a mathematical model incorporating the entraining effects of photic Zeitgebers on the core, the shell, and eventually the HPA axis. Our model reproduces a series of experimental observations including (1) the phase relation under $12 \; \mathrm{h}$/$12 \; \mathrm{h}$ light/dark entrainment; (2) temporal signal transient across the core, the shell, and the HPA axis; (3) a variety in individual’s ability to adapt to perturbed schedules such as jet lag and shift work. We predict that the individualized circadian adaption tolerances to jet lag and shift work are highly distinct among the population. Far from being coincidental, we found that an increased rhythmic flexibility results from a higher level of light sensitivity and AVP coupling strength. Thus, our work provides insights into the individualized allostatic adaptation strategies and functional trade-offs by investigating complex physiological entrainment architectures between the SCN and the HPA axis.

## Results

### General model structure

**Figure 1:**
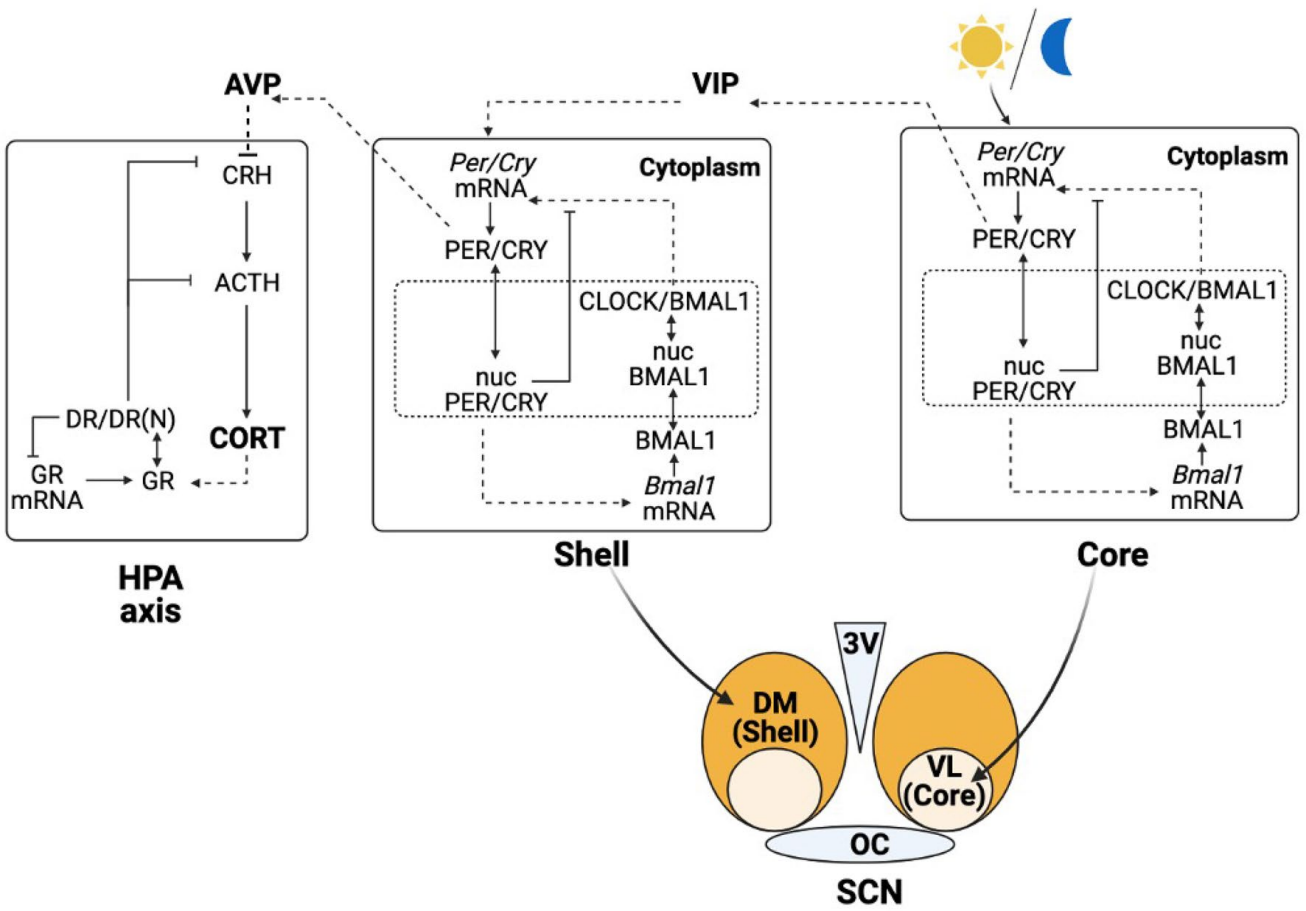
Schematic representation of the two-level model. Upstream, the positive and negative feedback of the SCN clock genes induce central rhythms entrained to the light/dark cycles. Downstream, the circadian oscillations of AVP as an output from the SCN entrain the HPA axis, leading to circadian oscillation of corticosterone (CORT).

To simulate synchronization within the SCN and between the SCN and the HPA axis, our model is constructed in two levels. At the SCN level, we use a single-cell gene regulatory network (GRN) model for neurons in both the core and the shell to describe the intracellular circadian dynamics of clock genes and proteins. The coupling within the SCN is accomplished by VIP neurotransmitters released from the core. At the HPA axis level, we consider the corticosterone dynamics which are regulated by the AVP signal released from the shell. The overall schematic of the model is shown in Fig. 1.

### Intracellular oscillators in the SCN

The entrainment dynamics of neurons in the SCN are based on an interlocked circadian oscillator model in mammals^20–22^. Neurons in the SCN core and shell share the same transcriptional and translational feedback structure which is described by seven ordinary differential equations. The intracellular network incorporates a positive and a negative feedback loop through which the SCN neurons exhibit self-sustained oscillations (Fig. 1; details described in “Materials and methods”).

### Synchronization of the core and the shell

To entrain the biological clock in the SCN to the photic Zeitgeber, light/dark cycles are modeled as a simple step function (“Materials and methods”), which is active for 12 h during the light period between Zeitgeber time (ZT) $0{-}12\; \text{h}$ and inactive during the dark period between ZT $12{-}24 \; \mathrm{h}$. The circadian release of neuropeptides mediates intercellular synchronization of oscillators in the SCN. We assume that the coupling between the core and the shell is accomplished by the neurotransmitter VIP upon PER/CRY protein activity as suggested by previous studies^23,24^. The releasing of VIP activates *Per/Cry* transcription in both the SCN core and shell, therefore functions as an entrainer for the non-photosensitive shell.

### Corticosterone regulation by AVP

The mathematical model for the HPA axis accounts for the stimulatory and negative feedback loop among CRH, ACTH, and corticosterone (CORT). The network is based on the skeletal structure of the Goodwin oscillator, previously proposed by Sriram et al.^25^ and later modified by Rao et al.^26^ to encompass the synchronization of the HPA axis activities by light/dark cycle via the SCN in both nocturnal and diurnal species (this study focuses on nocturnal species). In addition, we account for the synchronization effect of AVP on the HPA axis. Experiments demonstrated that AVP micro-infusions to rodents’ hypothalamus led to notable downregulation of adrenal corticosterone^27^. Moreover, shell-mediated secretion of AVP is crucial for the maintenance of PVN neuronal firing^28,29^, indicating that the autonomous oscillations of the HPA axis are entrained by the SCN, where AVP plays a crucial role. Therefore, we hypothesize that AVP, as a neurotransmitter output from the SCN, entrains the circadian profile of corticosterone by regulating the degradation of the PVN’s output, CRH.

### Model calibration

Twelve newly introduced parameters associated with light entraining signal, coupling effects of neurotransmitters VIP and AVP, and dynamic rates of VIP and AVP were estimated independently (denoted by ‘*’ in Table 1), while the remaining parameters were set to earlier determined values^21,26,30^, but slightly adjusted by scaling factors. The main attempt was to limit the parameter adjustment to avoid potential overfitting. This parameter estimation methodology allows to modify the system properties such as phase, period, and amplitude while maintaining the overall relation among the existing parameters. The parameter estimation aimed at satisfying the following criteria: (i) in constant-dark condition, the three compartments must be synchronized (i.e., the periods of different compartments are identical); (ii) when exposed to a $12\; \text{h}$/$12\; \text{h}$ light/dark entrainer (Zeitgeber), the system must be entrained (i.e., the periods of different compartments equal to $24\; \text{h}$); (iii) the intrinsic periods of the SCN core and shell should be in qualitative agreement with the experimental observations where the intrinsic period of the shell is less than the core, and both periods should not exceed $24\; \text{h}$; and (iv) the model should capture the correct phase relation among key oscillatory components: *Per/Cry* mRNA (core and shell), *Bmal1* mRNA (core and shell), VIP, AVP, and CORT. To account for coupling effects across light, the core, the shell, and the HPA axis, we use three coupling parameters ${v}_{l}$, ${v}_{c2}$ and ${v}_{coe}$ which represents light sensitivity of the core, the coupling strength of VIP, and the coupling strength of AVP, respectively (see “Materials and methods”). Since these three parameters couple the three independent compartments (the core, the shell, and the HPA axis), we define intrinsic periods of the three compartments as their periods in the absence of coupling (i.e., ${v}_{l}$, ${v}_{c2},$ ${v}_{coe}=0$). The estimation assigned the core, the shell, and the HPA axis intrinsic periods of $23.8\; \text{h}$, $21.2\; \text{h}$, $24.2\; \text{h}$, respectively. The 8% shorter period of the shell compared to the core qualitatively agrees with experimental findings^31^. Besides, the model reproduced a light-induced phase delay between the core and the shell of about $3\; \text{h}$ as observed experimentally^11^. The nominal parameter values and descriptions are included in Table 1. We assume that the nominal parameter set represents the optimum calibration state for our model.

**Table 1:** Model parameter values and their source.

#	Parameter	Value	Unit	Compartment/references	
1	${v}_{1bm}$	9.18	$\mathrm{nM}\; \text{h}^{-1}$	Core^30^	Maximal rate of Per/Cry transcription
2	${k}_{1bm}$	1.02	$\mathrm{nM}$		Michaelis constant of Per/Cry transcription
3	${k}_{1im}$	0.57	$\mathrm{nM}$		Inhibition constant of Per/Cry transcription
4	${v}_{c1}(*)$	0.1	$1$		VIP coupling strength in the core
5	${c}_{m}(*)$	1	$1$		Hill coefficient of activation effect of VIP
6	${p}_{m}$	8	$1$		Hill coefficient of inhibition of Per/Cry transcription
7	${k}_{1dm}$	0.12	$\text{h}^{-1}$		Degradation rate of Per/Cry mRNA
8	${v}_{l}(*)$	0.25	$1$		Light sensitivity in the core
9	${K}_{l}(*)$	50	$1$		Michaelis constant of light input
10	${k}_{2bm}$	0.31	${\mathrm{nM}}^{-1}\; \text{h}^{-1}$		Complex formation rate of Per-Cry mRNA
11	${q}_{m}$	2	$1$		Number of PER-CRY complex forming subunits
12	${k}_{2dm}$	0.05	$\text{h}^{-1}$		Degradation rate of cytoplasmatic PER-CRY
13	${k}_{2tm}$	0.24	$\text{h}^{-1}$		Nuclear import rate of the PER-CRY complex
14	${k}_{3tm}$	0.02	$\text{h}^{-1}$		Nuclear export rate of PER-CRY complex
15	${k}_{3dm}$	0.12	$\text{h}^{-1}$		Degradation rate of the nuclear PER-CRY complex
16	${v}_{4bm}$	3.67	$\mathrm{nM}\; \text{h}^{-1}$		Maximal rate of Bmal1 transcription
17	${k}_{4bm}$	2.2	$\text{nM}^{3}$		Michaelis constant of Bmal1 transcription
18	${r}_{m}$	3	$1$		Hill coefficient of activation of Bmal1 transcription
19	${k}_{4dm}$	0.77	$\text{h}^{-1}$		Degradation rate of Bmal1 mRNA
20	${k}_{5bm}$	0.24	$\text{h}^{-1}$		Translation rate of BMAL1
21	${k}_{5dm}$	0.06	$\text{h}^{-1}$		Degradation rate of cytoplasmatic BMAL1
22	${k}_{5tm}$	0.46	$\text{h}^{-1}$		Nuclear import rate of BMAL1
23	${k}_{6tm}$	0.06	$\text{h}^{-1}$		Nuclear export rate of BMAL1
24	${k}_{6dm}$	0.12	$\text{h}^{-1}$		Degradation rate of nuclear BMAL1
25	${k}_{6pm}$	0.09	$\text{h}^{-1}$		Activation rate of nuclear CLOCK-BMAL1
26	${k}_{7pm}$	0.003	$\text{h}^{-1}$		Deactivation rate of CLOCK-BMAL1
27	${k}_{7dm}$	0.01	$\text{h}^{-1}$		Degradation rate of CLOCK-BMAL1
28	${k}_{vs1}(*)$	1.5	$\text{h}^{-1}$		Synthesis rate of VIP
29	${k}_{dv1}(*)$	1.5	$\text{h}^{-1}$		Degradation rate of VIP
30	${v}_{1be}$	10.26	$\text{nM}\; \text{h}^{-1}$		Maximal rate of Per/Cry transcription
31	${k}_{1be}$	1.14	$\text{nM}$	Shell^30^	Michaelis constant of Per/Cry transcription
32	${k}_{1ie}$	0.64	$\text{nM}$		Inhibition constant of Per/Cry transcription
33	${v}_{c2}(*)$	1.01	$\text{nM}^{-2}$		VIP coupling strength in the shell
34	${c}_{e}(*)$	3	$1$		Hill coefficient of activation effect of VIP
35	${p}_{e}$	9	$1$		Hill coefficient of inhibition of Per/Cry transcription
36	${k}_{1de}$	0.14	$\text{h}^{-1}$		Degradation rate of Per/Cry mRNA
37	${k}_{2be}$	0.34	$\text{nM}^{-1}\; \text{h}^{-1}$		Complex formation rate of Per-Cry mRNA
38	${q}_{e}$	2	$1$		Number of PER-CRY complex forming subunits
39	${k}_{2de}$	0.06	$\text{h}^{-1}$		Degradation rate of cytoplasmatic PER-CRY
40	${k}_{2te}$	0.27	$\text{h}^{-1}$		Nuclear import rate of the PER-CRY complex
41	${k}_{3te}$	0.02	$\text{h}^{-1}$		Nuclear export rate of PER-CRY complex
42	${k}_{3de}$	0.13	$\text{h}^{-1}$		Degradation rate of the nuclear PER-CRY complex
43	${v}_{4be}$	4.1	$\text{nM}\; \text{h}^{-1}$		Maximal rate of Bmal1 transcription
44	${k}_{4be}$	2.46	$\text{nM}^{3}$		Michaelis constant of Bmal1 transcription
45	${r}_{e}$	3	$1$		Hill coefficient of activation of Bmal1 transcription
46	${k}_{4de}$	0.85	$\text{h}^{-1}$		Degradation rate of Bmal1 mRNA
47	${k}_{5be}$	0.27	$\text{h}^{-1}$		Translation rate of BMAL1
48	${k}_{5de}$	0.07	$\text{h}^{-1}$		Degradation rate of cytoplasmatic BMAL1
49	${k}_{5te}$	0.51	$\text{h}^{-1}$		Nuclear import rate of BMAL1
50	${k}_{6te}$	0.07	$\text{h}^{-1}$		Nuclear export rate of BMAL1
51	${k}_{6de}$	0.14	$\text{h}^{-1}$		Degradation rate of nuclear BMAL1
52	${k}_{6pe}$	0.1	$\text{h}^{-1}$		Activation rate of nuclear CLOCK-BMAL1
53	${k}_{7pe}$	0.003	$\text{h}^{-1}$		Deactivation rate of CLOCK-BMAL1
54	${k}_{7de}$	0.01	$\text{h}^{-1}$		Degradation rate of CLOCK-BMAL1
55	${k}_{vs2}(*)$	1	$\text{h}^{-1}$		Synthesis rate of AVP
56	${k}_{dv2}(*)$	1	$\text{h}^{-1}$		Degradation rate of AVP
57	${k}_{p1}$	0.38	$\upmu \text{M}\; \text{h}^{-1}$		Zero order synthesis rate constant of CRH
58	${K}_{p1}$	6.54	$\upmu \text{M}$	HPA axis^26^	Hypothalamic negative feedback
59	${V}_{d1}$	0.35	$\upmu \text{M}\; \text{h}^{-1}$		First order rate constant for CRH degradation
60	${K}_{d1}$	4.39	$\upmu \text{M}$		Michaelis–Menten constant for CRH degradation
61	${v}_{coe}(*)$	0.85	$1$		AVP coupling strength of the HPA
62	$s(*)$	3	$1$		Hill coefficient of activation effect of AVP
63	${k}_{p2}$	0.46	$\upmu \text{M}\; \text{h}^{-1}$		First order rate constant for synthesis of ACTH
64	${K}_{p2}$	1.63	$\upmu \text{M}$		Pituitary negative feedback
65	${V}_{d2}$	1	$\upmu \text{M}\; \text{h}^{-1}$		First order rate constant for degradation of ACTH
66	${K}_{d2}$	0.85	$\upmu \text{M}$		Michaelis–Menten constant for ACTH degradation
67	${k}_{p3}$	0.73	$\text{h}^{-1}$		Feedforward adrenal sensitivity
68	${V}_{d3}$	0.72	$\upmu \text{M} \; \text{h}^{-1}$		First order rate constant for CORT degradation
69	${K}_{d3}$	0.18	$\upmu \text{M}$		Michaelis–Menten constant for CORT degradation
70	${k}_{syn,GRm}$	2.9	$\text{fmolg}^{-1}\; \text{h}^{-1}$		Zero order rate constant for synthesis of GR mRNA
71	${k}_{syn,GR}$	1.2	$GR\left(0\right) \; {k}_{deg,GR}/GRm(0)$		First order rate constant for degradation of GR
72	${r}_{f}$	0.49	$1$		GR recycle fraction from nucleus to cytoplasm
73	${k}_{re}$	0.57	$\text{h}^{-1}$		Rate of GR recycling from nucleus to cytoplasm
74	${k}_{on}$	0.03	$\text{L} \; \text{nmol}^{-1}\; \text{h}^{-1}$		Second-order rate constant for CORT-GR binding
75	${k}_{deg,GR}$	0.06	${\mathrm{h}}^{-1}$		First order rate constant for degradation of GR
76	${k}_{deg,GRm}$	0.11	${k}_{syn,GRm}/GRm(0)$		First order rate constant for degradation of GR mRNA
77	${k}_{T}$	0.63	$\text{h}^{-1}$		Rate of GR translocation from cytoplasm to nucleus

Parameters estimated in this study are denoted with a (*).

**Figure 2:**
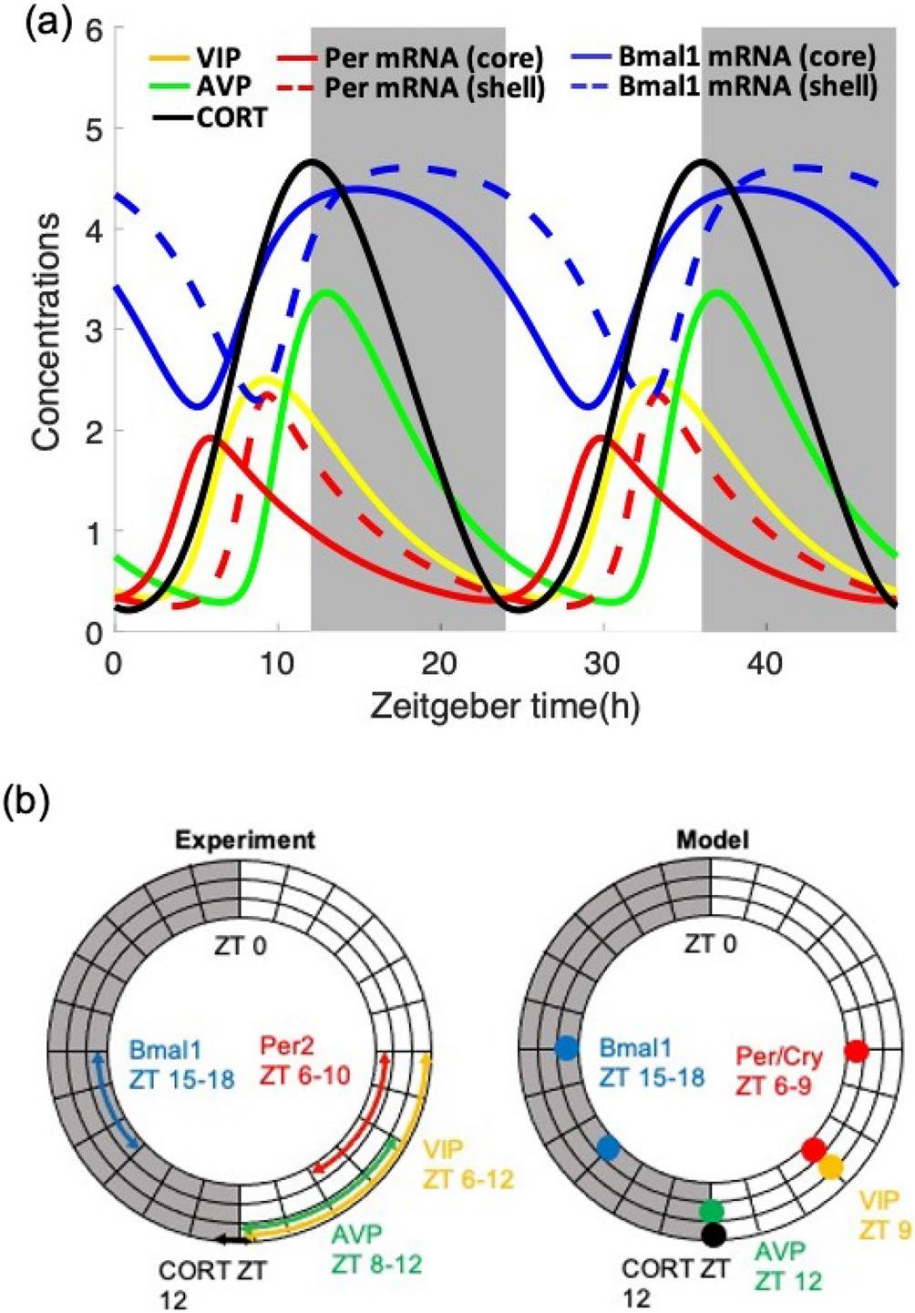
The interactions between the SCN and the HPA axis orchestrate the physiological response to the photic Zeitgeber. (**a**) Dynamic profiles of clock gene mRNAs, VIP, AVP, and CORT generated by the model with nominal parameters as indicated in Table 1. All components oscillate with a period of 24 h once the system gets entrained. *Bmal1* mRNA oscillates almost anti-phasic to *Per/Cry* mRNA. mRNAs in the shell oscillate with a phase delay of 3 h compared to mRNAs in the core. White/dark patterns denote light/dark settings. (**b**) Experimentally determined phase relation between the representative components (left) and model predictions (right) are consistent^63–66^.

Figure 2a depicts the dynamics of seven representative components (clock genes’ mRNA, neurotransmitters, and corticosterone) as the model gets entrained by a rhythmic light/dark stimulus ($L12$/$D12$). Entrainment drives the SCN and the HPA axis autonomous rhythms to adopt period of the Zeitgeber (light), and as a result, all the components’ phases track the light/dark cycles (Fig. 2b). Specifically, the almost anti-phasic relation between *Bmal1* and *Per/Cry* mRNA is reproduced by the model, driven by the activated form of BMAL1^20^. The peaking time of the clock genes’ mRNA in the shell exhibits a time delay compared to the core since the expression of PER/CRY in the core drives the secretion of VIP, which ultimately entrains the shell. Experimental evidence suggests *Per1* mRNA expression in shell lags that of the core by about an hour, following a light pulse^11^. This feature is qualitatively captured by our model (albeit predicting a slightly greater delay of about $3\; \text{h}$), suggesting the sequential information transduction within the SCN from the core to the shell. Similarly, VIP and AVP exhibit a phase delay as VIP peaks earlier during the subjective day, while AVP peaks around dusk. In the HPA axis, corticosterone secretion reaches a maximum at the onset of the dark period, which is the beginning of the active phase for nocturnal species.

**Figure 3:**
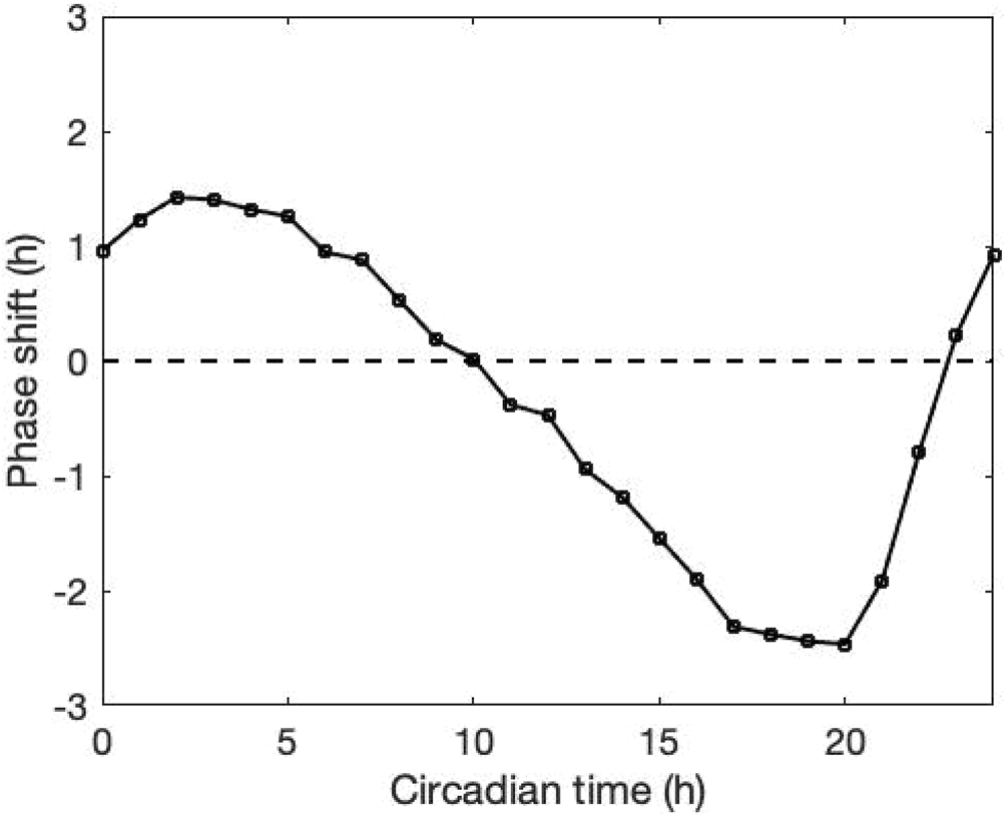
Phase curve response. Light stimulus with 3 h duration was applied at different times of corticosterone free-running cycle. Dashed line represents the non-phase-shift condition. Light stimulus advanced circadian rhythms when delivered during the subjective day (CT 0–10) and late subjective night (CT 23–24). During the early subjective night (CT 11–22), light stimulus delayed the rhythm.

Since light is the key entrainer, we evaluated the model’s response to light by determining the phase response curve (PRC). A $3\; \text{h}$ light stimulus nearly $12$ times higher than the rhythmic light intensity was introduced at different subjective time, after the system had acclimated to constant darkness (DD). Since in DD there is no light/dark Zeitgeber time (ZT), we defined the point where corticosterone peaks as circadian time twelve (CT12), following experiments setting the subjective time to twelve at the beginning of the nocturnal animal’s active phase. Figure 3 depicts the PRC by measuring the phase changes between the perturbed and unperturbed corticosterone profiles. The resulting PRC is of *type I*, where both phase advance and phase delay can be produced continuously depending upon the timing of the photic perturbation. Phase delay is observed when the light is introduced at the descending phase of CORT (between CT 11 and CT 22). In experimental observations, a typical PRC exhibits phase delay in the early subjective night and phase advance in the late subjective night, with little phase-shifting occurring during the subjective day. Hence, the subjective day portion of the PRC is often referred to as the "dead zone"^32^. In our model, light stimulus causes phase delay when introduced from CT 11 to CT 22, while a slight phase advance is predicted when light pulses are introduced during the early subjective day and late subjective night. Therefore, our model predicts a large delay-to-advance ratio, which is consistent with experimental observations^33^.

### Model synchronization dynamics

**Figure 4:**
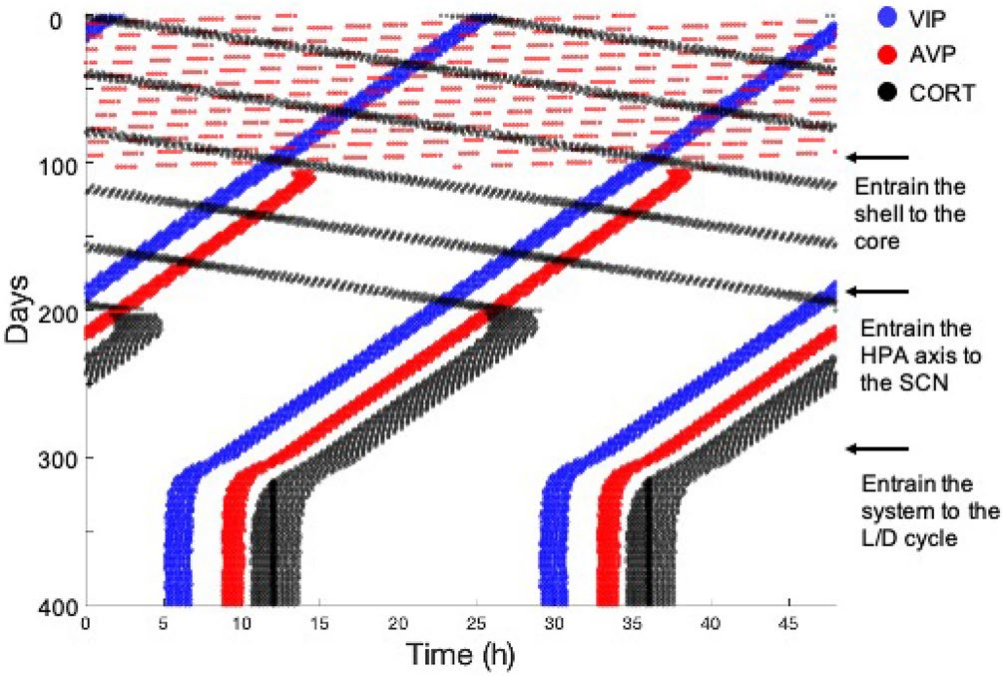
Simulated actograms of the coupled three compartments model showing the synchronization dynamics of the system. The actogram is constructed when the levels of the component’s oscillation are above their corresponding thresholds. We define the threshold as minimum value of the component’s oscillation profile plus 0.9 times of its amplitude value. Blue, red, and black horizontal lines represent peaking activities of VIP, AVP, and CORT as well as the corresponding compartments: the core, the shell, and the HPA axis. Arrows on the right indicate the time when coupling parameters get varied. At the beginning, when the coupling sensitivities are absent, the nonparallel phase patterns indicate the uncoupling status among different compartments. With the three coupling parameters being restored to their nominal values, both internal synchronization and external entraining occur step by step. Eventually, all the components maintain a stable phase relation and oscillate with a 24 h period.

An essential and innovative feature of the model is the existence of coupling links across different independent compartments. Understanding the synchronization mechanisms of the system as well as the role of the three coupling parameters is critical to revealing the underlying physiological relations. In our work, we focus on VIP, AVP, and CORT as the key outputs to represent the three compartments: the core, the shell, and the HPA axis, respectively. It is worth mentioning that VIP and AVP are not only outputs of their corresponding compartments but also entrainers to the subsequent compartment. The computational “actograms” of VIP, AVP, and CORT show the synchronization procedure of our system (Fig. 4). As shown in the beginning stage of the actograms, the SCN core and shell, as well as the HPA axis, oscillate independently with their intrinsic periods with the absence of the three coupling parameters (i.e., ${v}_{l}= 0, {v}_{c2}=0, {v}_{coe}=0$). As the uncoupled system gets to steady-state, VIP coupling strength is firstly restored to its nominal value, resulting in the synchronization of AVP indicated by the parallel phase patterns of VIP and AVP. Subsequently, AVP coupling strength is restored to its nominal value after the new steady-state has been reached, resulting in the synchronization of CORT. Finally, with the re-establishment of light sensitivity, VIP, AVP, and CORT get entrained by the light/dark cycle, indicated by the vertical phase patterns with a $24\; \text{h}$ period oscillations. These results clearly illustrate the synchronization dynamics of the system, demonstrating the regulation and entrainment of the inner compartments by the external Zeitgeber.

**Figure 5:**
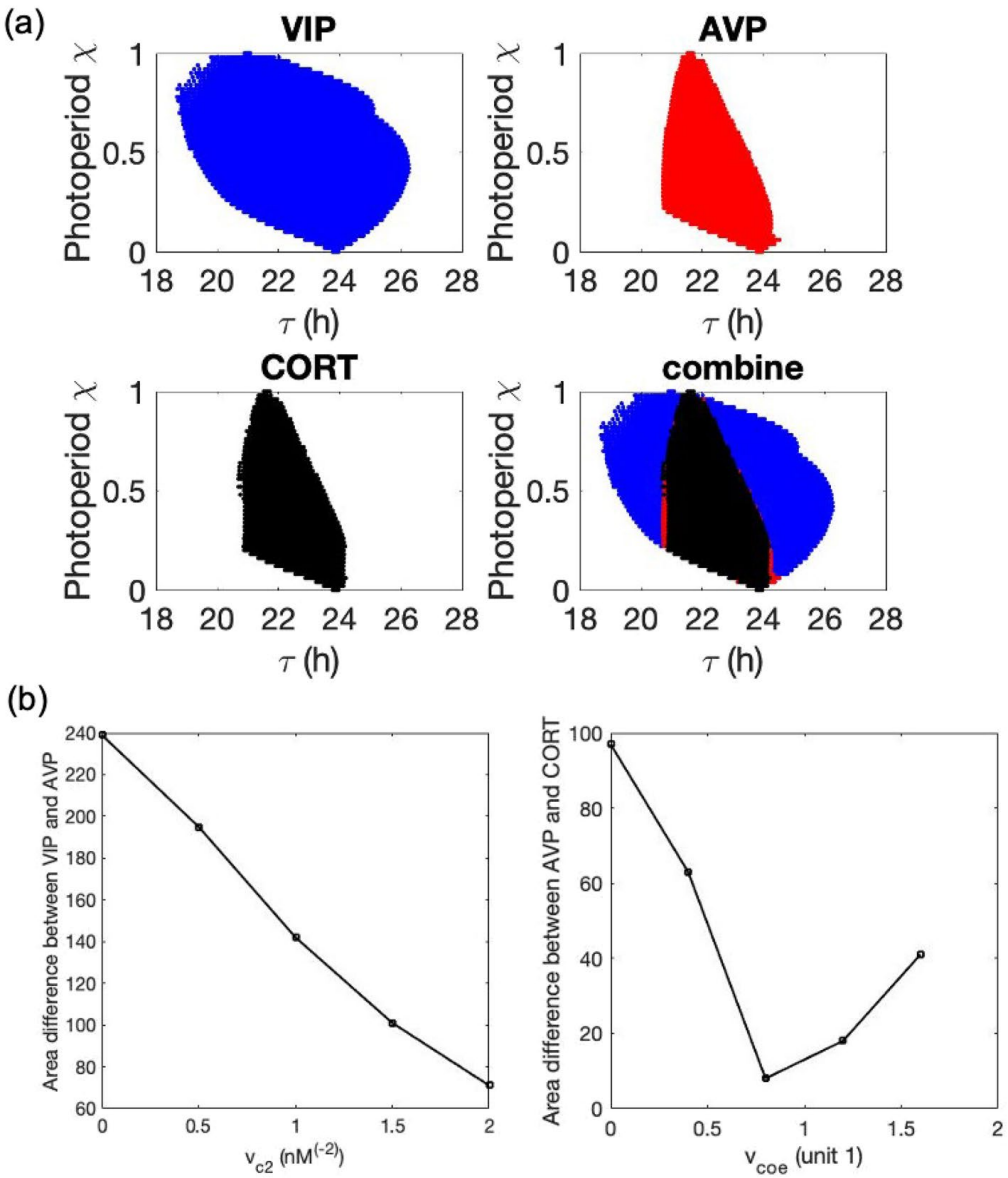
Entrainment ranges of the representative output of the three compartments. We set the Zeitgeber as a step function with different periods where the photoperiod $\chi$ is defined as the fraction of the light phase. (**a**) Arnold onions in the $\chi -\tau$ plane given a Zeitgeber strength. Blue, red, and black patterns denote the entrainment range of VIP, AVP and CORT variables, representing the core, the shell, and the HPA axis compartments. (**b**) The area differences between VIP and AVP, AVP and CORT as function of corresponding coupling parameters v_c2_ and v_coe_.

Next, we investigated the entrainment range differences (i.e., the period mismatch between the subsystems and the Zeitgeber that leads to entrainment) among the three compartments, and more importantly, how the values of the coupling parameters affect the entrainment. To evaluate the entrainment range of the system, we adopted the protocol suggested by Schmal et al.^34^, whereby the entrainment status is characterized on the “Zeitgeber period – Zeitgeber photoperiods” parameter plane. Within the entrainment region, Zeitgeber enforces its period in the circadian compartments so that the compartments get entrained. The resulting entrainment zones for AVP, VIP, and CORT are shaped in the form of skewed “*Arnold onions*”, Fig. 5a. For each Arnold onion, the bottom and upper points are affected by the free-running periods in constant-dark (DD) and constant-light (LL), respectively. The tips (photoperiod $\chi =0 \; or \;1$) of the onion pointing to $Zeitgeber \;period{\text{-}}axis$ are given by periods of the system under DD (${\uptau }_{\mathrm{DD}}$) and LL (${\uptau }_{\mathrm{LL}}$), respectively. We determined that the Arnold onions are skewed toward the left since the system shortens its period with increasing light intensity. The widest entrainment range is found near the equinoctial photoperiod (i.e., $Zeitgeber \; photoperiod \; \chi =0.5$). As intuitively expected, the entrainment range diminishes moving from the core to the shell and eventually to the periphery (i.e., $\mathrm{VIP}>\mathrm{AVP}>\mathrm{CORT}$). Area difference between the Arnold onions of two neighboring compartments represents the entraining ability of one over the other, where a smaller area difference corresponds to a better entraining ability. We determined the area differences between VIP and AVP, AVP and CORT as a function of the corresponding coupling strength values. As shown in Fig. 5b, as the coupling strength of VIP (i.e., ${v}_{c2}$) increases, the entraining ability of VIP over the shell increases, while as the coupling strength of AVP (i.e., ${v}_{coe}$) increases, the entraining ability of AVP over the HPA axis firstly increase and then decreases. The non-monotonic profile of the coupling effect might be due to the nonlinear nature of the circadian oscillators. More details related to the effects of coupling strengths will be discussed later.

### In-silico population and their response to perturbed light schedules

**Figure 6:**
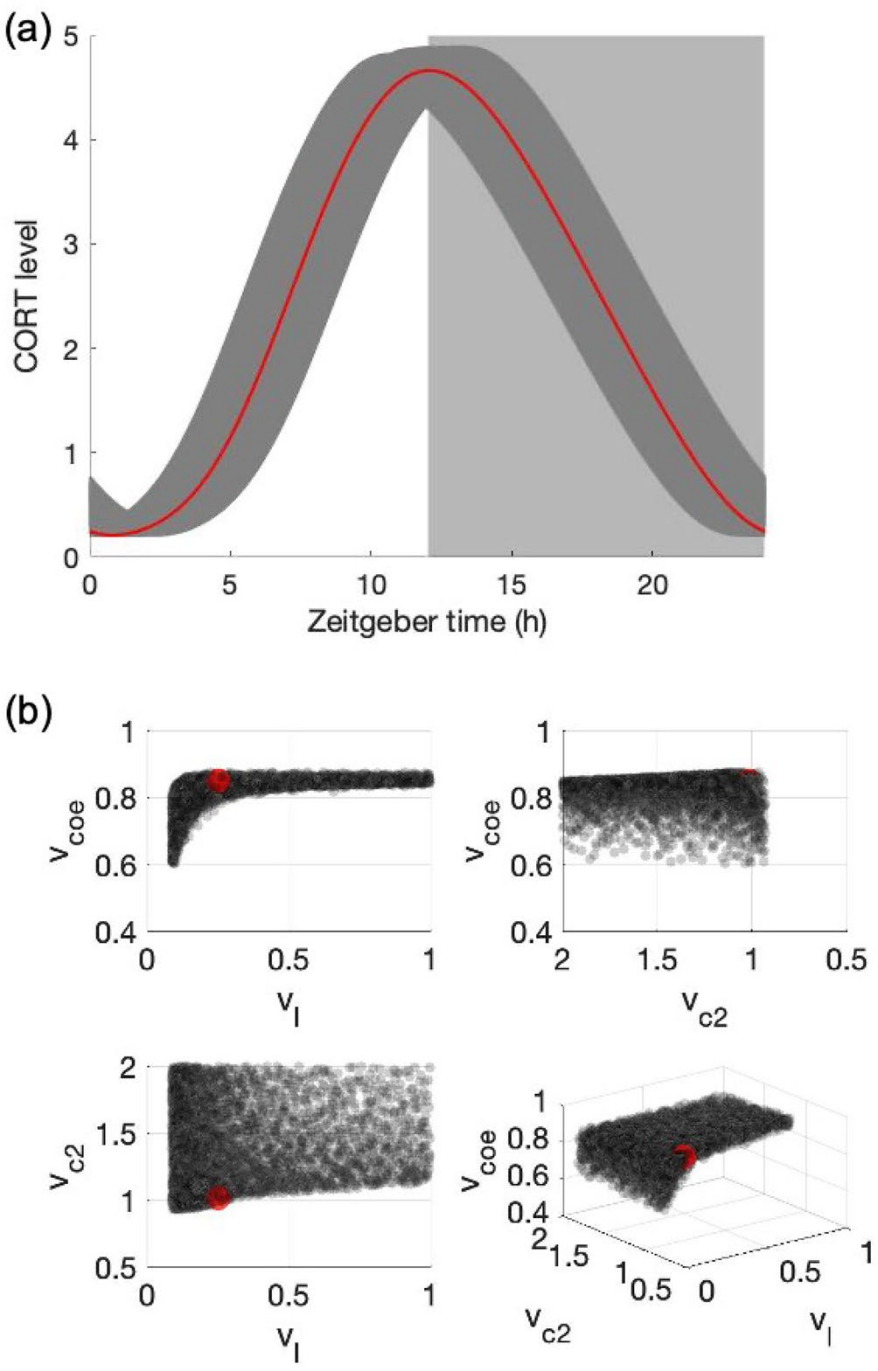
(**a**) Simulated corticosterone profiles of the selected in-silico population. Red curve refers to the nominal CORT profile, while dark-gray shadow refers to the range where other sampling CORT profiles lie in. White/light-grey square patterns denote the light/dark cycle. (**b**) selected parameter space where the circadian profiles of the simulated CORT lie within ± 20% of the nominal CORT profile. Gray dots refer to the virtual individuals, and red dot refers to the nominal parameter set. There is a monotonically increasing balance between v_l_ and v_coe_ that as v_l_ increases, v_coe_ increases in a relatively narrow range. The main, left, and above views show the existence of thresholds for v_l_ and v_c2_ such that they generate a robust coupled system that matches the experimental rhythms of CORT.

As the primary outcome of the HPA axis, the CORT circadian phenotype should be maintained within strict physiological bounds to support downstream homeostatic mechanisms. However, significant individual variability exists within these physiological bounds, and this difference potentially contributes to personalized synchronization strategies that allow for differential responses to transient and permanent light schedule changes. We hypothesize that individual synchronization is driven by, among others, the inter-individual differences in coupling parameters within the SCN and between the SCN and the HPA axis as reflected in parameters ${v}_{l}$, ${v}_{c2}$ and ${v}_{coe}$. These parameters reflect physiologically crucial light sensitivity and the coupling strengths of VIP and AVP. The interplay between these values results in synchronization heterogeneity in a compensatory manner to maintain CORT circadian rhythms within reasonable bounds. A virtual population was created using Sobol sampling^35,36^ where the distribution of three coupling parameters was identified such that the circadian profiles of simulated CORT lie within ± 20% of the nominal CORT profile (Fig. 6a). This sampling approach helps to determine the subspace of three parameters (${v}_{l}$, ${v}_{c2}$ and ${v}_{coe}$) that corresponds to homeostatic CORT rhythms. We find that ${v}_{l}$ and ${v}_{c2}$ have relatively wide acceptable distribution ranges while the range of ${v}_{coe}$ values is narrow (Fig. 6b). This selection result is consistent with our observation in Fig. 5b, since the increase of ${v}_{l}$ and ${v}_{c2}$ leads to a stronger entraining effect for the SCN core and shell, while for ${v}_{coe}$, only a narrow range of its value induces strong coupling effects of the HPA axis.

Jet lag and shift work increase the incidence of chronic pathologies, such as diabetes, depression, and cancer, driven by the temporal misalignment of the endogenous circadian oscillations with the external Zeitgebers. Previous studies^26,37^ have shown pronounced individual differences in jet lag and shift work tolerance, while the correlation between jet lag tolerance and shift work tolerance is less studied. To better understand the inter-individual differences in response to perturbed light schedules and the underlying trade-offs between the ability to adapt to jet lag and shift work, we evaluated the capacity of the system to respond to perturbations in light schedules reflecting various forms of jet lag and shift work.

**Figure 7:**
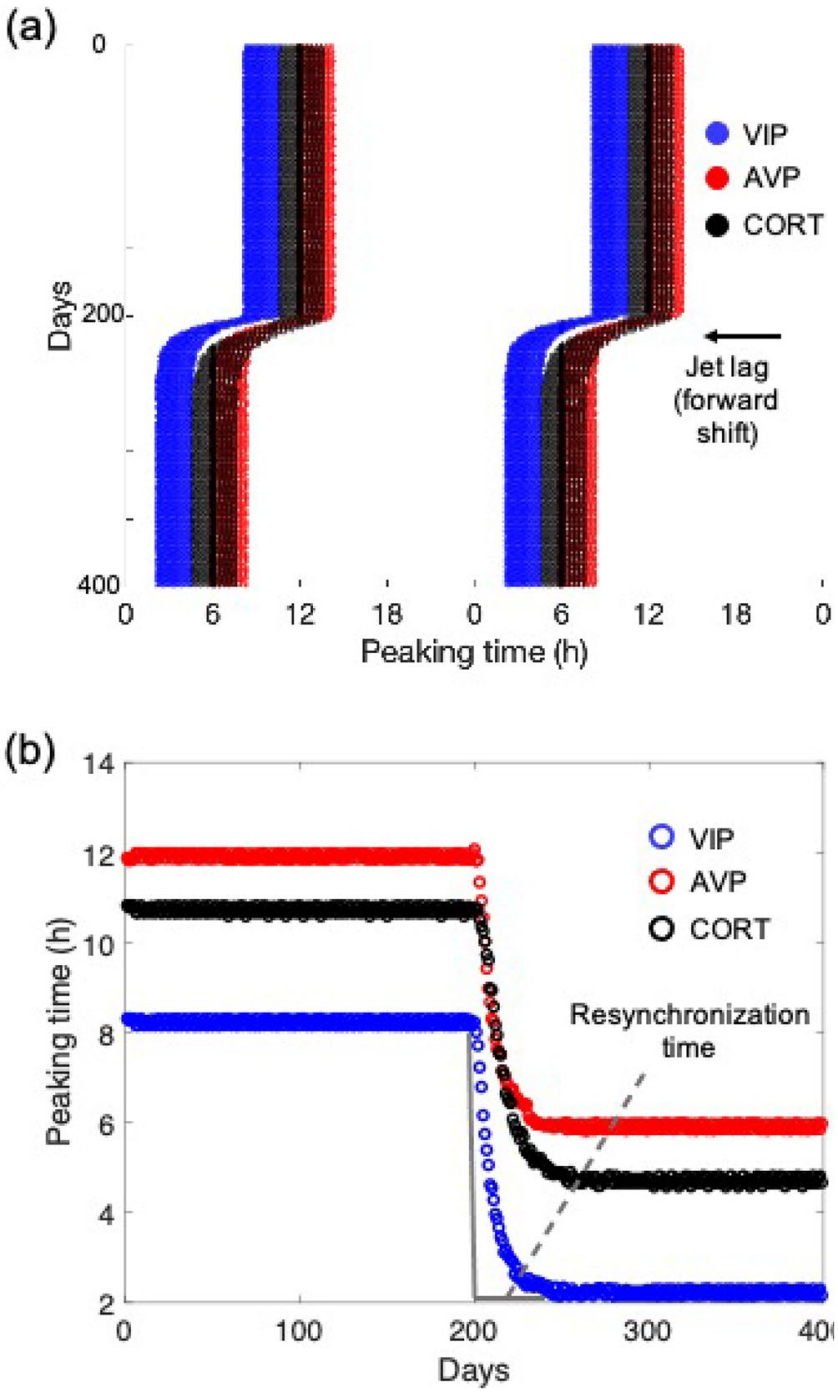
Resynchronization dynamics when exposed to jet lag schedule. (**a**) Representative double-plotted actogram of VIP, AVP, and CORT before and after 6-h LD phase advance applied at day 200. (**b**) Peaking phase shifts of VIP, AVP, and CORT under jet lag. Resynchronization time was defined as the time at which phase shift was completed.

**Figure 8:**
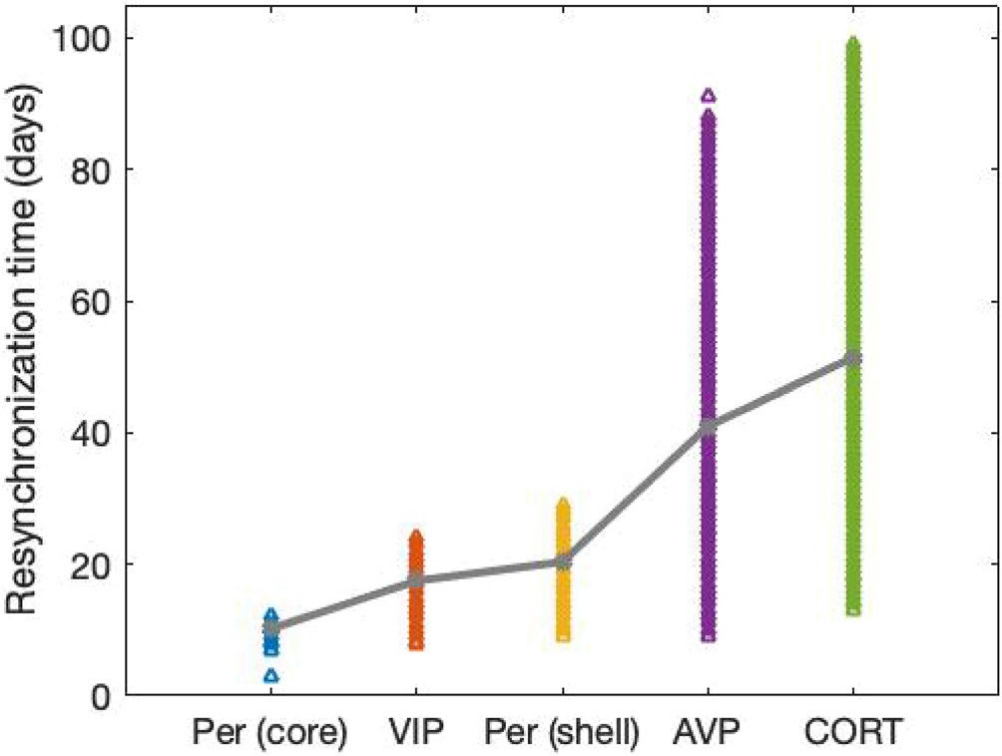
The resynchronization time of Per/Cry mRNA in the core and the shell, VIP, AVP, and CORT for all the selected individuals are denoted by triangle signs. The mean values of the five components are represented by gray dots.

To characterize the response of the model to jet lag, we first conducted a jet lag simulation under the nominal parameter set. A $6\; \text{h}$ advanced light phase shift (simulating eastward traveling) is introduced once the host has been acclimated to a regular light schedule. The change in light schedule triggered a gradual shift of the peaking time of VIP, AVP, and CORT towards the new light regimen (Fig. 7). Eventually, the system reaches a new steady-state characterized by stable $24\; \text{h}$ oscillation with a $6\; \text{h}$ phase advance. The phase shift is most rapid at the early stages of the transitions and then decreases exponentially (Fig. 7b). Our model predictions are consistent with experimental observations^38^. We consider a component to be resynchronized when its phase shift is larger than $5.8\; \text{h}$. By testing the resynchronization time of 5 components (*Per/Cry* mRNA in the core, VIP, *Per/Cry* mRNA in the shell, AVP, CORT) among all the selected individuals, we observed an increase in resynchronization time as the entraining signal transitions from upstream to downstream, resulting in a minimum resynchronization time of Per/Cry mRNA in the core and a maximum resynchronization time of CORT in the HPA axis (Fig. 8). Analogous findings were reported by Kiessling et al.^39^, where a strong heterogeneity in resynchronization time under jet lag schedule was found between different organs and tissues following an entraining signal transduction order from the SCN to the adrenal, and then to the peripheral cells. Our model qualitatively captured this adaptation heterogeneity between different compartments due to the hierarchical structure of the physiological oscillatory system.

**Figure 9:**
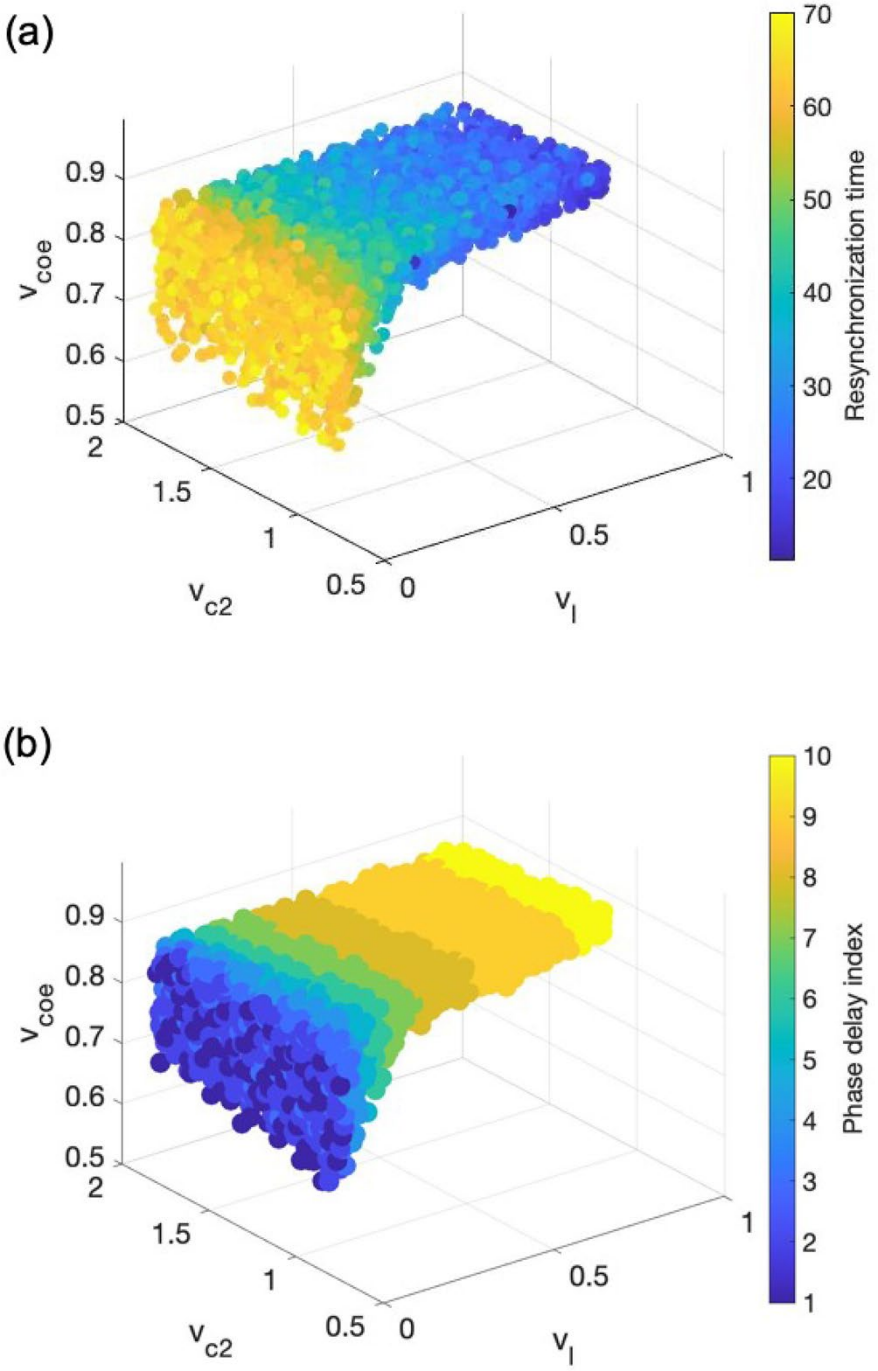
The shifting direction and adaptation rate of the system on exposure to jet lag. (**a**) The time to resynchronization (unit: day) in CORT rhythm was determined upon a permanent, abrupt inversion (12 h reversed) in the light/dark schedule. The resynchronization time decreases with light sensitivity increases. (**b**) The distribution of the phase delay index (dimensionless) among the simulated population. The color depicts the individual’s ability to exhibit phase delay. Individuals with higher light sensitivity have a stronger capacity to adapt to phase delay shifting jet lag.

Adrenal glucocorticoids play a key role in circadian re-entrainment under jet lag and shift work. Manipulation of the phase-shifting of adrenal glucocorticoid circadian rhythms regulates the speed of behavioral re-entrainment. Therefore, we focused our characterization on the CORT when investigating inter-individual responses to jet lag and shift work. Figure 9a records the resynchronization time of CORT for all the selected individuals under a $12\; \text{h}$ jet lag perturbation. As light sensitivity and APV coupling strength (${v}_{l}$ and ${v}_{coe}$) increase, the time to resynchronization decreases, indicating that individuals who are more sensitive to light and AVP bear a better jet lag adaptation ability. The inter-individual diversity in jet lag resynchronization time is distinctive, with more than a threefold difference among the simulated population. Another intriguing feature is the direction of jet lag. Experimental studies have shown that eastward (phase advance) travel is typically worse than westward (phase delay) travel for human beings, due to the fact that the average intrinsic period of the circadian clock in humans is longer than $24\; \text{h}$^40^. Similarly, the simulated system shows a stronger tendency to exhibit phase advances than phase delays since its intrinsic period is shorter than $24\; \text{h}$. To quantify the ability of each individual to exhibit phase delay in jet lag, we recorded the CORT shifting direction for each individual under $23$ shifting schedules $(+1 \; \mathrm{ h}$, $+2 \; \mathrm{h}$, …, $+12 \; \mathrm{ h}$, $-1 \; \mathrm{h}$, $-2 \; \mathrm{h}$, …, $-11 \; \mathrm{ h};$ "$+$" means phase advance schedule while "$-$" means phase delay schedule). We define the phase delay index as the number of the schedules under which CORT exhibits phase delay shifting (rightward in actograms). Our results show that the maximum value of phase delay index is 10, which is smaller than the overall phase delay schedule numbers (which is 11), meaning all the virtual individuals have a stronger tendency to shift advancingly. The level of this tendency varies among the population and is mainly affected by the light sensitivity and AVP coupling strength (Fig. 9b). Moreover, an individual’s ability to exhibit phase delay shift is positively related to its resynchronization rate under jet lag, suggesting the existence of the inner correlation between the resynchronization direction and adaptation rate.

**Figure 10:**
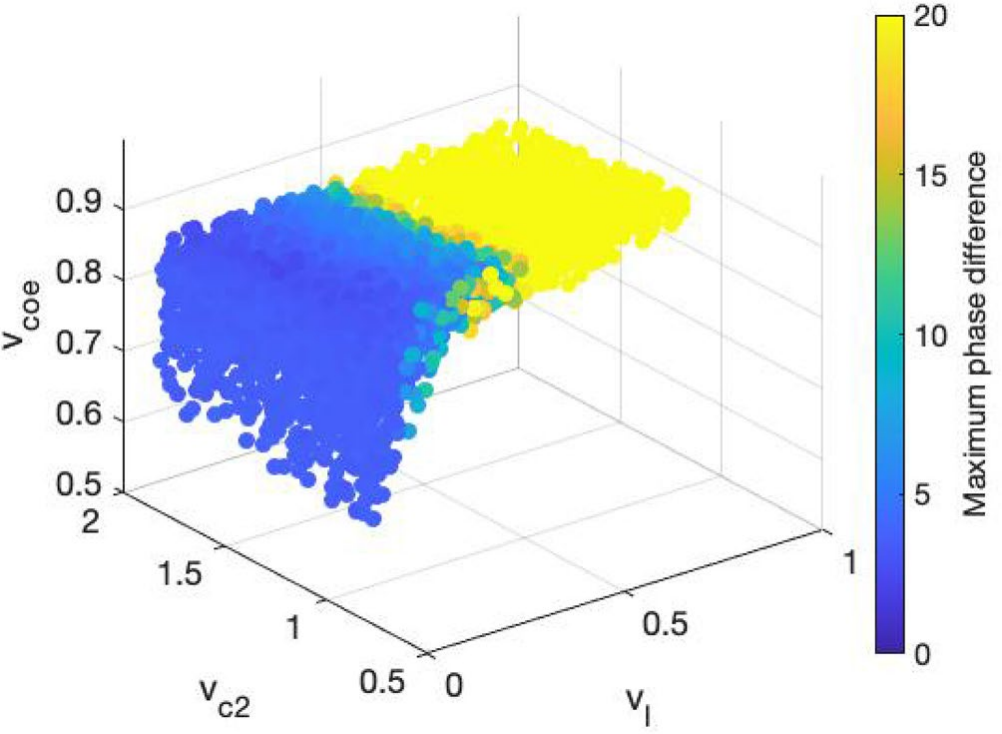
The response of the system to transient shift work. CORT’s peaking phase was measured upon a transient inversion in the light/dark schedule lasting for 8 days. The maximal phase shift (unit: h) increases with increasing light sensitivity. This implies that simulated individuals with higher light sensitivity are more vulnerable to transient shift work.

To understand both long-term and short-term effects of shift work, we simulated two types of shift work schedules: transient shift work and permanent alternating shift work. The former denotes a temporary perturbation^26^ in which an $8\text{-day}$ transient inversion in the light/dark schedule was introduced. The maximum phase difference between perturbed and unperturbed CORT oscillations was measured as a gauge of tolerance to short term shift work (a larger phase difference represents a lower level of tolerance). Once again, our model predicts a parametric dependence of the transient shift work tolerance (Fig. 10). Specifically, a higher level of light sensitivity and AVP coupling strength leads to a lower level of transient shift work tolerance. Combining these with the jet lag findings, our model predicts that those individuals whose circadian clock adapts more efficiently to jet lag are more susceptible to transient shift work. This trade-off was also indicated in our earlier work^26^.

**Figure 11:**
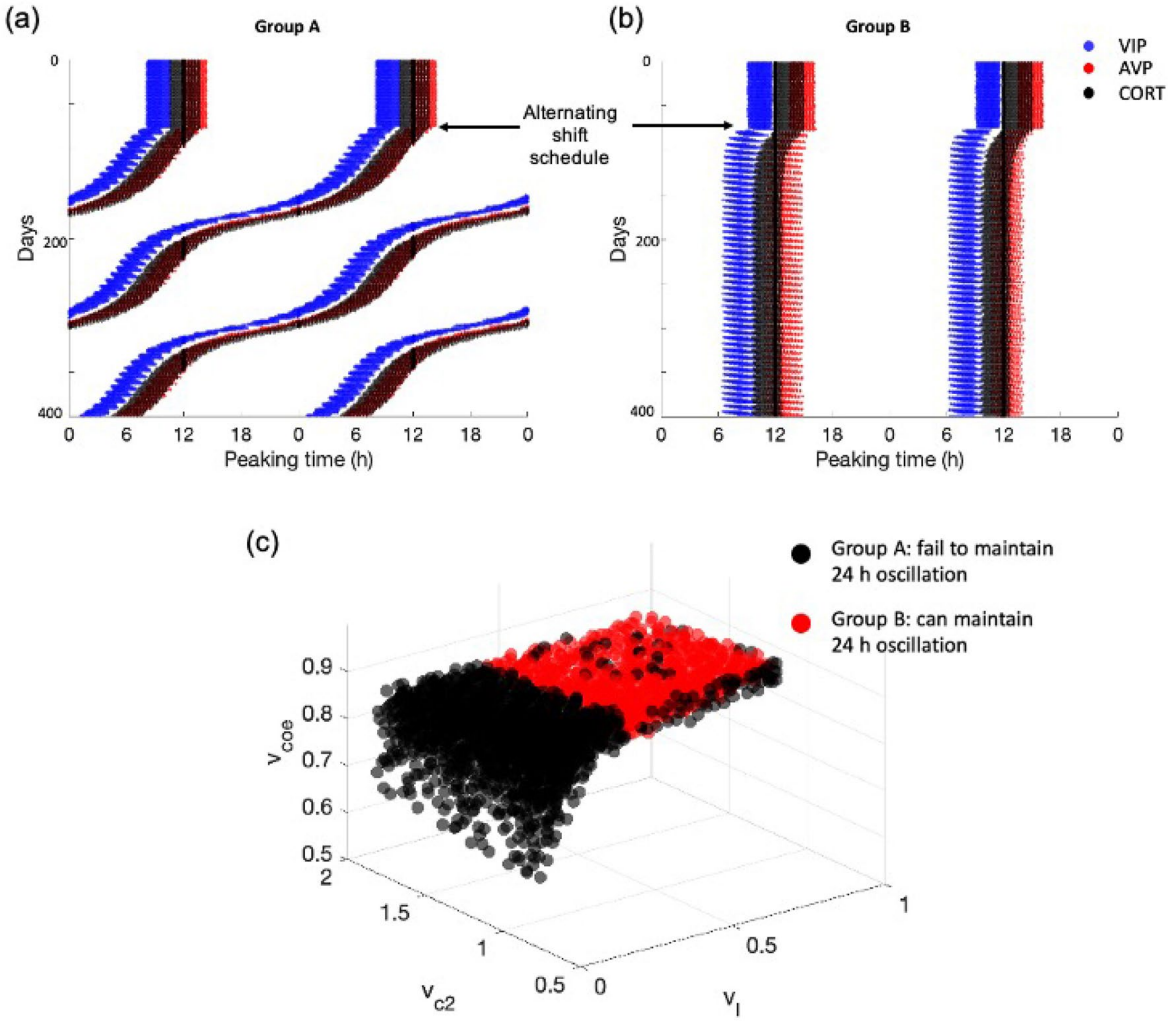
The response of the system to alternating shift work. The system was subjected under a 2-day inverse/5-day normal fast-rotating schedule after 75 days. Two types of response were observed. (**a**) Representative peaking time actograms for individuals who fail to maintain a stable 24 h oscillation; (**b**) Representative peaking time actograms for individuals who are able to maintain a stable 24 h oscillation. (**c**) The parameter space distribution of the two groups. All the individuals from group A are concentrated at high level of light sensitivity, while individuals from group B are mainly distributed at lower light sensitivity area, with a wider distribution compared to group A.

Following this, we sought to determine the effects of alternating shift work patterns. We examined the system response to rotating light/dark cycles with $2\; \text{days}$ inversion every $7\; \text{days}$ (essentially simulating a 7-day schedule: $5\; \text{days}$ normal and $2\; \text{days}$ reversed shift). Following exposure to alternating shift work schedule, a distinct inter-individual variation was observed (Fig. 11a,b). Based on whether an individual can maintain their period within $24 \; \text{h} \pm 0.05 \; \text{h}$ or not, the population was assigned in one of the two groups. Figures 11a,b represent the actograms upon exposure to the fast-rotating schedule for individuals who failed (group A) and succeeded (group B) to maintain a $24\; \text{h}$ oscillation. Individuals in group A appear to manifest a “free running” status given that their period is smaller than $24\; \text{h}$. Individuals in group B show a greater propensity to maintain the normal light/dark cycle. While individuals in group B adapt, their phases are slightly shifted compared to their initial phases. The phase-locked behavior in alternating shift work of group B was also reported in experimental observations and was hypothesized to serve as a mechanism in the long term to prevent detrimental consequences of oscillations having to repeatedly resynchronize to alternating patterns of shift work^41^. Therefore, we hypothesize that individuals belonging to group B can tolerate the fast-rotating schedule better than individuals in group A. The parametric space distributions of the two groups are shown in Fig. 11c, where individuals from group A are denoted by black dots while individuals from group B are marked by red dots. Our results show that in general, individuals from group A have a smaller level of light sensitivity and AVP coupling strength compared to those from group B. Combining the previous results, we found that tolerance to alternating shift work and transient shift work are inversely correlated. This inter-correlation indicates that the better tolerance of group B upon the exposure to the alternating shift work may be due to the benefits of its faster transient rate to the alternating schedules.

**Figure 12:**
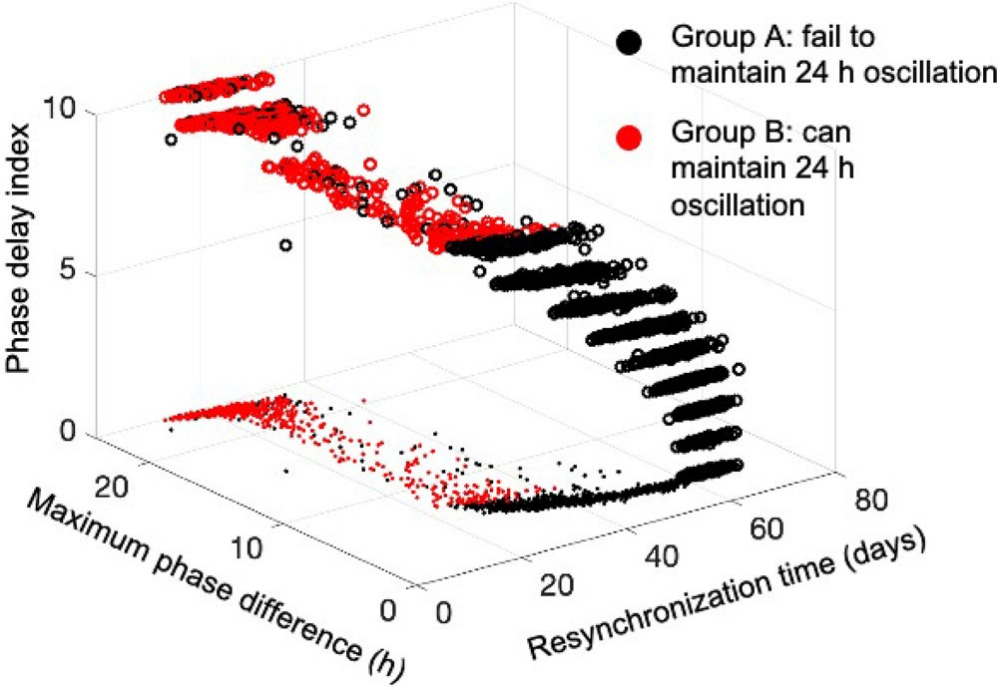
Correlations among system responses to different perturbed circadian schedules. The tolerance to reversed jet lag (qualified by resynchronization time), the ability to follow the external direction shifting direction of jet lag (qualified by phase delay index), and the tolerance to alternating shift work (grouped by red and black colors) are positively correlated to each other, while these three tolerances and the transient shift work tolerance are negatively correlated.

So far, we have established a map of the effects of the synchronization within the SCN and between the SCN and the HPA axis as it relates to the emergence of circadian flexibility under different schedules of shift work and jet lag, where the relations between different entrainment properties are shown in Fig. 12. Our results indicate that (i) an individual’s ability to shift in the same direction as the outside Zeitgeber changes, (ii) an individual’s tolerance to jet lag, and (iii) an individual’s tolerance to alternating shift work are positively correlated with each other, showing a consistency in shift work and jet lag tolerances. We define the regulatory plasticity of individuals from group B to be higher than those from group A, since individuals from group B have a shorter resynchronization time and can adapt to the external shifting directions more easily. This plasticity also accounts for the trade-offs between the tolerance to transient shift work and the alternating shift work, since a lower tolerance of the former and a higher tolerance of the latter both result from the emergence of a higher regulatory plasticity.

## Discussion

Entrainment of the internal physiological clock by the external environment is essential to maintaining homeostasis. As the central effector that controls organismal behavior, the HPA axis conveys entrainment signals from the master pacemaker (SCN) to peripheral cells. When light/dark schedules become irregular, the SCN output to the HPA axis becomes disturbed, resulting in glucocorticoids rhythm, phase and period disruptions which have been linked to numerous pathologies^18^. The complexity of the circadian timing in our body requires a systematic approach to investigate a multitude of interactions. While previous mathematical models have incorporated the entraining effects of light on the HPA axis^21,22,26,36^, to our knowledge, this study is one of the first attempts to construct an integrated multi-compartment model combing the heterogeneous light entrainment of the SCN core and shell, and the subsequent entrainment of the HPA activity through efferent output signals from the SCN.

Our model explored the temporal dynamic and light responses for the three compartments: the SCN core and shell, and the HPA axis. We observed the existence of temporal information transfer across these compartments under regular (Fig. 2) and perturbed light/dark schedules (Figs. 7 and 8). Under a regular schedule, the components’ peaking phases follow a spatially and temporally ordered pattern, as entrainment signals transduce from the environment (light input) through the core to the shell, and eventually the HPA axis. Between the core and the shell, there is a time lag for the light-induced *Per* expression to spread since neurons in the core project to the shell, but not the reverse^12^ suggesting that temporal information is transmitted from the core to the shell^42^. In agreement with jet lag experiments^39^, our model predicts considerable transient desynchronization of the system, where the adaptation time-delay varies moving from the SCN core to the shell and eventually the HPA axis. The simulated alternating circadian phase shift also causes desynchronization among different oscillators (Fig. 11a,b). These misalignments among different compartments are likely to cause detrimental effects related to jet lag and shift work.

We hypothesize that the differences in coupling parameters values (defined by ${v}_{l}$, ${v}_{c2}$ and ${v}_{coe}$) define the inter-individual regulatory plasticity. Our results indicate the existence of a balance between the three regulatory mechanisms to match the desired corticosterone levels, Fig. 6b. While AVP coupling strength lies within tight bounds, as indicated by the narrow distribution of ${v}_{coe}$ values, both light sensitivity and VIP coupling strength show high variability among individuals, with the establishment of their threshold values as indicated in Fig. 6b (${v}_{l}$, $> 0.1$, ${v}_{c2} > 0.8$). Such high diversity in light sensitivity has also been experimentally observed with more than a 50-fold difference across individuals^43^. In addition to stimulating the visual system, light elicits responses related to physiological and neurobehavioral functions, known as the non-image forming (NIF) effects of the light^44^. Light sensitivity has a critical effect on NIF functions, such as the circadian entrainment, and shifts the timing of circadian rhythms. Previous experiments have found a potential consistency in the tolerance to disrupted schedules, as both male and young groups were reported to have an improved ability to cope with shift work and jet lag compared to female and aging groups^45,46^. Our model suggests that the physiological cause of this consistency may be related to the coupling strengths. A lower light sensitivity has been reported among female and aging groups^47,48^. On the other hand, earlier studies have shown that chronic jet lag increases mortality in aged mice, and aging groups have difficulty recovering from simulated jet lag^49,50^, while social jet lag is more detrimental to female in their cognitive ability compared to male^51^, and female may struggle more with the effects of jet lag or night shifts than male^52^. Our model predictions show that individuals with lower light sensitivity are more vulnerable to shift work and take longer to recover from jet lag, implying that the attenuation of circadian entrainment in jet lag and shift work among aging or female groups may be caused by their lower light sensitivity. In other words, inter-individual differences in light sensitivity may explain differential vulnerability to circadian disruption and subsequent impact on human health. Based on this, our model suggests that light therapy could be used to improve circadian entrainment under perturbed schedule among vulnerable population.

Apart from light sensitivity, VIP coupling strength is also essential in maintaining intracellular and behavioral rhythmicity in the SCN. Studies have shown that mice bearing a null mutation of the VIP receptors cannot maintain normal circadian rhythms. Moreover, mice lacking VIP in the SCN have abnormal circadian activity and reduced synchrony among neurons^53^. Although VIP is also expressed through out other parts of the mammalian brain such as cortex, retina, and superior colliculus, a recent study by Mazuski et al. shows that VIP from the SCN is crucial in sustaining daily rhythms synchronization. Therefore, VIP has been hypothesized to be necessary for the intra-SCN connectivity and generation of the SCN output to hypothalamic areas^54^. In accord with the implications of these findings, our model predicts that keeping VIP coupling strength above a minimum value inside the SCN is crucial for maintaining the rhythmicity throughout the system. The insufficiency of VIP coupling strength can be due to a lack of VIP neurons or VIP receptors. Furthermore, our model also predicts that the role of VIP coupling strength plays in perturbed circadian schedules is negligible since there is almost no variation in response of the system as ${v}_{c2}$ changes (Figs. 9, 10 and 11). Earlier work has shown that AVP released by neurons of the SCN strongly affects the depressive disorder among patients. Once over-expressed or over-released, AVP may contribute to hyper-anxiety and disturbed rhythmicity^55^. Our model incorporates the effect of AVP levels and consistent with the experiments, our results suggest a narrow range for AVP coupling strength to maintain normal behavior homeostasis. More importantly, we predict that, within this range, AVP coupling strength increases as light sensitivity increases. Both light sensitivity and AVP coupling strength significantly affect an individual's flexibility when exposed to perturbed schedules.

We explored the system response under non-rhythmic constant darkness condition (Fig. 3). We tested the PRC as a probe for understanding the circadian regulatory mechanism of the system. As a *type I* PRC, the system displays relatively small phase shifts with a continuous transition between phase delays and advances. The phase-resetting indicated by the PRC curve compensates for the fact that the free-running period of the system is not equal to $24\; \text{h}$. In other words, light can reset the clock to equalize the period of the entrained oscillator to the period of its cycle. With the verified entrainment ability of the system, we further investigated the difference in the ability to get entrained by measuring the area of Arnold onions for three compartments (Fig. 5). The left-skewed Arnold onions indicate that constant light induces a decrease in the intrinsic period of the system. This prediction does not abide by Aschoff’s first rule in which higher light intensity is associated with longer circadian periods in nocturnal organisms. This disobedience may be due to the missing of one pathway in our model where constant light lengthens the nocturnal circadian period by elevating levels of mPER2 protein and constitutively enhance the phase-delaying limb of the molecular oscillator^56^. Our model also predicts a coupling parametric dependence of entrainment ranges. Specifically, the entrainment range of VIP and AVP gets increased as their corresponding coupling sensitivity ${v}_{l}$ and ${v}_{c2}$ gets larger, while the entrainment range of CORT firstly increases then decreases as ${v}_{coe}$ increases with the maximal achieved at ${v}_{coe}\approx 0.8$. The parametric dependencies shown in Fig. 5b accounts for the parameter sets distribution after selection shown in Fig. 6b, since the prerequisite for individuals to fit in the nominal CORT phenotype is to establish a well coupled and entrained multi-compartment system.

Finally, we explored inter-individual adaptation mechanisms in perturbed schedules. Studies show that during long-term shift work, complete adjustment is seldom achieved^38^. By categorizing the simulated population into a susceptible group (group A) and a resistant group (group B), our results also indicate the lack of entertainment for both group A and B in alternating shift work schedules. Individuals from group A oscillate with an unstable period smaller than $24\; \text{h}$, while although individuals from group B establish a stable $24\; \text{h}$ period, they also fail to completely track the external rotating schedule. By tracking the previous steady light/dark schedule, group B establishes a mechanism in the long term to prevent the maladaptive consequences of rhythms having to constantly re-entrain to a rotating pattern of shift work. Thus, though both groups A and B failed to be entrained by the rotating schedule, resistant individuals exhibit a less pronounced de-synchronization. Because the susceptible individuals are also less prone to exhibit phase delay shifts in jet lag (Fig. 9b), we hypothesize that their higher susceptibility to alternating shift work mainly results from their stronger propensity to shift in the same direction as their intrinsic period (i.e., phase advance). This tendency to align their rhythm to their free-running pattern also contributes to a lesser phase shift in transient shift work of group A. Overall, our model predicts that individuals with higher light sensitivity tend to have enhanced ability to cope with jet lag and alternating shift work, but not transient shift work. The mathematical model of the SCN and the HPA axis that we used in this study has several limitations. For instance, the model failed to reproduce the “dead zone” part of the phase response curve due to the fact that the system didn’t include the phase-dependent sensitivity (gating) effects^30^. Moreover, it is well understood that apart from its classic circadian oscillation, the HPA axis also exhibits prominent ultradian pattern of discrete pulsatile release of glucocorticoids. Apart from that, glucocorticoids have also been shown to have feedback effects on central circadian clocks, adding complexity to the circadian dynamics of glucocorticoids. Nevertheless, studies have shown that many essential properties of glucocorticoid rhythms can be explained using circadian limit cycle oscillators of the HPA axis^25,26,36,57–59^. Therefore, with future modifications of the model that gradually incorporate the mentioned properties, we expect that the key features of our simulation results might be preserved.

In summary, our model describes the molecular rhythms observed in the SCN and the HPA axis and describes the entrainment pathways at multiple levels. Focusing on the coupling parameters between the three compartments (SCN core, SCN shell, and the HPA axis), we investigated the likely drivers of individualized susceptibility under different circadian perturbations, proposing a better interpretation of the relation between the genotypic diversity and reactivity of a population composed of phenotypically similar individuals. Since the model incorporates the essential structure and processes of the circadian system, future work includes the multi-cell scale models describing the synchronization of neurons in the SCN; the introduction of other Zeitgebers such as sleep and metabolism; the inclusion of more parameters to account for inter-individual variety.

## Materials and methods

### SCN compartment

In the clock network of both the core and shell, the nuclear translocated PER/CRY proteins (y_3_ and y_11_) downregulate their synthesis by stimulating *Bmal1* gene transcription (positive feedback) and inhibiting the activity of the CLOCK/BMAL1 heterocomplex (negative feedback). The heterocomplex CLOCK/BMAL1 (y_7_ and y_15_) functions as an activator for the transcription of *Per/Cry* genes (y_1_ and y_8_). In the positive feedback loop, after the transcription of Per/Cry mRNA, the expressed PER/CRY proteins (y_2_ and y_10_) translocate to the nucleus (y_3_ and y_11_). The PER/CRY proteins in the nucleus activate *Bmal1* mRNA (y_4_ and y_12_) transcription, which further increases the expression of CLOCK/BMAL1 heterodimer (y_7_ and y_15_) after the translation to BMAl1 protein (y_5_ and y_13_) and the protein translocation to the nucleus (y_6_ and y_14_). In the negative feedback loop, PER/CRY proteins in the nucleus shut off the transcriptional activity of the CLOCK/BMAL1 heterocomplex. The positive and negative feedback structures essentially result in the autonomous oscillators. The behavior of the clock components in the core and the shell is described by Eqs. (2–8) and (10–16), respectively.

Light/dark cycles are modeled as a L12/D12 step function (Eq. 1). We introduce the light effect by an additional saturation of photoreceptors term in Eq. (2) encompassing the light-induced transcriptional activation of *Per/Cry* mRNA in the light-sensitive core. This additive term accounts for the independence of photic-induced transcription from CLOCK/BMAL1 driven transcription^60^. The Michaelis–Menten equation is used to account for the saturation of photoreceptors^61^.

To ensure that the neurotransmitter is released quickly after *Per/Cry* mRNA transcription, we assume the neurotransmitters are released upon cytosolic PER/CRY protein activity as suggested by other modeling works^23,24^. Correspondingly, VIP is induced by PER/CRY protein in the core (Eq. 9), while AVP is induced by PER/CRY protein in the shell (Eq. 17). The coupling between neurons in the core and the shell is accomplished by the neurotransmitter VIP, which leads to *Per/Cry* mRNA transcription in both the core and the shell (Eq. 2 and 10). In the VIP coupling terms, Hill coefficients are used to control the steepness of the Per/Cry promoter feedback loop.

1 $$\mathrm{light}=\{1, 0\le \mathrm{ZT}<12 0, 12\le \mathrm{ZT}<24$$

2 $$\frac{\mathrm{dPer}/{\mathrm{Cry}}_{\mathrm{mRNA}}(\mathrm{core})}{\mathrm{dt}}=\frac{{\mathrm{v}}_{1\mathrm{bm}}\left(\mathrm{CLOCK}/\mathrm{BMAL}1(\mathrm{core})+{\mathrm{v}}_{\mathrm{c}1} \cdot {\mathrm{VIP}}^{\mathrm{cm}}\right)}{{\mathrm{k}}_{1\mathrm{bm}}\left(1 +{\left(\frac{\mathrm{nucPER}/\mathrm{CRY}(\mathrm{core})}{{\mathrm{k}}_{1\mathrm{im}}}\right)}^{\mathrm{pm}}+\mathrm{CLOCK}/\mathrm{BMAL}1(\mathrm{core})+{\mathrm{v}}_{\mathrm{c}1} \cdot {\mathrm{VIP}}^{\mathrm{cm}}\right)}-{\mathrm{k}}_{1\mathrm{dm}} \cdot \mathrm{Per}/{\mathrm{Cry}}_{\mathrm{mRNA}}(\mathrm{core})+{\mathrm{v}}_{\mathrm{l}}\frac{\mathrm{light}}{\mathrm{light}+{\mathrm{K}}_{\mathrm{l}}}$$

3 $$\frac{\mathrm{dPER}/\mathrm{CRY}(\mathrm{core})}{\mathrm{dt}}={\mathrm{k}}_{2\mathrm{bm}}\cdot {{\mathrm{Per}/\mathrm{Cry}}_{\mathrm{mRNA}}(\mathrm{core})}^{\mathrm{qm}}-{\mathrm{k}}_{2\mathrm{dm}}\cdot \mathrm{PER}/\mathrm{CRY}(\mathrm{core})-{\mathrm{k}}_{2\mathrm{tm}}\cdot \mathrm{PER}/\mathrm{CRY}(\mathrm{core})+{\mathrm{k}}_{3\mathrm{tm}}\cdot \mathrm{nucPER}/\mathrm{CRY}(\mathrm{core})$$

4 $$\frac{\mathrm{dnucPER}/\mathrm{CRY}(\mathrm{core})}{\mathrm{dt}}={\mathrm{k}}_{2\mathrm{tm}}\cdot \mathrm{PER}/\mathrm{CRY}(\mathrm{core}) -{\mathrm{k}}_{3\mathrm{tm}}\cdot \mathrm{nucPER}/\mathrm{CRY}(\mathrm{core})-{\mathrm{k}}_{3\mathrm{dm}}\cdot \mathrm{nucPER}/\mathrm{CRY}(\mathrm{core})$$

5 $$\frac{\mathrm{dBmal}{1}_{\mathrm{mRNA}}(\mathrm{core})}{\mathrm{dt}}=\frac{{\upnu }_{4\mathrm{bm}}\cdot \mathrm{nucPER}/\mathrm{CR}{\mathrm{Y}(\mathrm{core})}^{\mathrm{rm}}}{{\mathrm{k}}_{4\mathrm{bm}}+\mathrm{nucPER}/\mathrm{CR}{\mathrm{Y}(\mathrm{core})}^{\mathrm{rm}}}-{\mathrm{k}}_{4\mathrm{dm}}\cdot \mathrm{Bmal}{1}_{\mathrm{mRNA}}(\mathrm{core})$$

6 $$\frac{\mathrm{dBMAL}1(\mathrm{core})}{\mathrm{dt}}={\mathrm{k}}_{5\mathrm{bm}}\cdot \mathrm{Bmal}{1}_{\mathrm{mRNA}}(\mathrm{core})-{\mathrm{k}}_{5\mathrm{dm}}\cdot \mathrm{BMAL}1(\mathrm{core})-{\mathrm{k}}_{5\mathrm{tm}}\cdot \mathrm{BMAL}1(\mathrm{core})+{\mathrm{k}}_{6\mathrm{tm}}\cdot \mathrm{nucBMAL}1(\mathrm{core})$$

7 $$\frac{\mathrm{dnucBMAL}1(\mathrm{core})}{\mathrm{dt}}={\mathrm{k}}_{5\mathrm{tm}}\cdot \mathrm{BMAL}1(\mathrm{core})-{\mathrm{k}}_{6\mathrm{tm}}\cdot \mathrm{nucBMAL}1(\mathrm{core})-{\mathrm{k}}_{6\mathrm{dm}}\cdot \mathrm{nucBMAL}1(\mathrm{core})+{\mathrm{k}}_{7\mathrm{pm}}\cdot \mathrm{CLOCK}/\mathrm{BMAL}1(\mathrm{core})-{\mathrm{k}}_{6\mathrm{pm}}\cdot \mathrm{nucBMAL}1(\mathrm{core})$$

8 $$\frac{\mathrm{dCLOCK}/\mathrm{BMAL}1(\mathrm{core})}{\mathrm{dt}}={\mathrm{k}}_{6\mathrm{pm}}\cdot \mathrm{nucBMAL}1(\mathrm{core})-{\mathrm{k}}_{7\mathrm{pm}}\cdot \mathrm{CLOCK}/\mathrm{BMAL}1(\mathrm{core})-{\mathrm{k}}_{7\mathrm{dm}}\cdot \mathrm{CLOCK}/\mathrm{BMAL}1(\mathrm{core})$$

9 $$\frac{\mathrm{dVIP}}{\mathrm{dt}}={\mathrm{k}}_{\mathrm{vs}1}\cdot \mathrm{PER}/\mathrm{CRY}(\mathrm{core})-{\mathrm{k}}_{\mathrm{dv}1}\cdot \mathrm{VIP}$$

10 $$\frac{\mathrm{dPer}/{\mathrm{Cry}}_{\mathrm{mRNA}}(\mathrm{shell})}{\mathrm{dt}}=\frac{{\mathrm{v}}_{1\mathrm{be}}\left(\mathrm{CLOCK}/\mathrm{BMAL}1(\mathrm{shell})+{\mathrm{v}}_{\mathrm{c}2} \cdot {\mathrm{VIP}}^{\mathrm{ce}}\right)}{{\mathrm{k}}_{1\mathrm{be}}\left(1 +{\left(\frac{\mathrm{nucPER}/\mathrm{CRY}(\mathrm{shell})}{{\mathrm{k}}_{1\mathrm{ie}}}\right)}^{\mathrm{pe}}+\mathrm{CLOCK}/\mathrm{BMAL}1(\mathrm{shell})+{\mathrm{v}}_{\mathrm{c}2} \cdot {\mathrm{VIP}}^{\mathrm{ce}}\right)}-{\mathrm{k}}_{1\mathrm{de}} \cdot \mathrm{Per}/{\mathrm{Cry}}_{\mathrm{mRNA}}(\mathrm{shell})$$

11 $$\frac{\mathrm{dPER}/\mathrm{CRY}(\mathrm{shell})}{\mathrm{dt}}={\mathrm{k}}_{2\mathrm{be}}\cdot {{\mathrm{Per}/\mathrm{Cry}}_{\mathrm{mRNA}}(\mathrm{shell})}^{\mathrm{qe}}-{\mathrm{k}}_{2\mathrm{de}}\cdot \mathrm{PER}/\mathrm{CRY}(\mathrm{shell})-{\mathrm{k}}_{2\mathrm{te}}\cdot \mathrm{PER}/\mathrm{CRY}(\mathrm{shell})+{\mathrm{k}}_{3\mathrm{te}}\cdot \mathrm{nucPER}/\mathrm{CRY}(\mathrm{shell})$$

12 $$\frac{\mathrm{dnucPER}/\mathrm{CRY}(\mathrm{shell})}{\mathrm{dt}}={\mathrm{k}}_{2\mathrm{te}}\cdot \mathrm{PER}/\mathrm{CRY}(\mathrm{shell}) -{\mathrm{k}}_{3\mathrm{te}}\cdot \mathrm{nucPER}/\mathrm{CRY}(\mathrm{shell})-{\mathrm{k}}_{3\mathrm{de}}\cdot \mathrm{nucPER}/\mathrm{CRY}(\mathrm{shell})$$

13 $$\frac{\mathrm{dBmal}{1}_{\mathrm{mRNA}}(\mathrm{shell})}{\mathrm{dt}}=\frac{{\upnu }_{4\mathrm{be}}\cdot \mathrm{nucPER}/\mathrm{CR}{\mathrm{Y}(\mathrm{shell})}^{\mathrm{re}}}{{\mathrm{k}}_{4\mathrm{be}}+\mathrm{nucPER}/\mathrm{CR}{\mathrm{Y}(\mathrm{shell})}^{\mathrm{re}}}-{\mathrm{k}}_{4\mathrm{de}}\cdot \mathrm{Bmal}{1}_{\mathrm{mRNA}}(\mathrm{shell})$$

14 $$\frac{\mathrm{dBMAL}1(\mathrm{shell})}{\mathrm{dt}}={\mathrm{k}}_{5\mathrm{be}}\cdot \mathrm{Bmal}{1}_{\mathrm{mRNA}}(\mathrm{shell})-{\mathrm{k}}_{5\mathrm{de}}\cdot \mathrm{BMAL}1(\mathrm{shell})-{\mathrm{k}}_{5\mathrm{te}}\cdot \mathrm{BMAL}1(\mathrm{shell})+{\mathrm{k}}_{6\mathrm{te}}\cdot \mathrm{nucBMAL}1(\mathrm{shell})$$

15 $$\frac{\mathrm{dnucBMAL}1(\mathrm{shell})}{\mathrm{dt}}={\mathrm{k}}_{5\mathrm{te}}\cdot \mathrm{BMAL}1(\mathrm{shell})-{\mathrm{k}}_{6\mathrm{te}}\cdot \mathrm{nucBMAL}1(\mathrm{shell})-{\mathrm{k}}_{6\mathrm{de}}\cdot \mathrm{nucBMAL}1(\mathrm{shell})+{\mathrm{k}}_{7\mathrm{pe}}\cdot \mathrm{CLOCK}/\mathrm{BMAL}1(\mathrm{shell})-{\mathrm{k}}_{6\mathrm{pe}}\cdot \mathrm{nucBMAL}1(\mathrm{shell})$$

16 $$\frac{\mathrm{dCLOCK}/\mathrm{BMAL}1(\mathrm{shell})}{\mathrm{dt}}={\mathrm{k}}_{6\mathrm{pe}}\cdot \mathrm{nucBMAL}1(\mathrm{shell})-{\mathrm{k}}_{7\mathrm{pe}}\cdot \mathrm{CLOCK}/\mathrm{BMAL}1(\mathrm{shell})-{\mathrm{k}}_{7\mathrm{de}}\cdot \mathrm{CLOCK}/\mathrm{BMAL}1(\mathrm{shell})$$

17 $$\frac{\mathrm{dAVP}}{\mathrm{dt}}={\mathrm{k}}_{\mathrm{vs}2}\cdot \mathrm{PER}/\mathrm{CRY}(\mathrm{shell})-{\mathrm{k}}_{\mathrm{dv}2}\cdot \mathrm{AVP}$$

### HPA axis compartment

In the HPA axis, CRH regulates the release of ACTH, which eventually induces the release of CORT (corticosterone in this work) (Eqs. 18–20). The model also considers the pharmacodynamics of the bound cortisol-receptor complex as adapted from Ramakrishnan et al.^62^ (Eqs. 21–24). In particular, the CORT receptor (GR) binds to CORT in the cytoplasm of the target cells, resulting in the formation of the receptor/CORT complex (DR) (Eq. 23). DR ultimately translocates to the nucleus (DR(N)) and is assumed to be responsible for the negative feedback effects of CORT on to CRH and ACTH.

For the coupling term, the AVP response of the HPA axis is further modulated through the saturation of AVP receptors (Eq. 18). The Hill coefficient denotes the cooperative character of the AVP regulation process. A lower Hill coefficient leads to a more gradual inhibition of the promoter, whereas a high Hill coefficient results more in a switch-like process^23^.

18 $$\frac{\mathrm{dCRH}}{\mathrm{dt}}=\frac{{\mathrm{k}}_{\mathrm{p}1}{\mathrm{K}}_{\mathrm{p}1}}{{\mathrm{K}}_{\mathrm{p}1}+\mathrm{DR}\left(\mathrm{N}\right)}-{\mathrm{V}}_{\mathrm{d}1} \cdot \frac{\mathrm{CRH}}{{\mathrm{K}}_{\mathrm{d}1}+\mathrm{CRH}} \cdot \left(1+{\mathrm{v}}_{\mathrm{coe}} \cdot \frac{{\mathrm{AVP}}^{\mathrm{s}}}{1+{\mathrm{AVP}}^{\mathrm{s}}}\right)$$

19 $$\frac{\mathrm{dACTH}}{\mathrm{dt}}=\frac{{\mathrm{k}}_{\mathrm{p}2}{\mathrm{K}}_{\mathrm{p}2}\mathrm{CRH}}{{\mathrm{K}}_{\mathrm{p}2}+\mathrm{DR}\left(\mathrm{N}\right)}-{\mathrm{V}}_{\mathrm{d}2}\frac{\mathrm{ACTH}}{{\mathrm{K}}_{\mathrm{d}2}+\mathrm{ACTH}}$$

20 $$\frac{\mathrm{dCORT}}{\mathrm{dt}}={\mathrm{k}}_{\mathrm{p}3} \cdot \mathrm{ACTH}-{\mathrm{V}}_{\mathrm{d}3}\frac{\mathrm{CORT}}{{\mathrm{K}}_{\mathrm{d}3}+\mathrm{CORT}}$$

21 $$\frac{{\mathrm{dGR}}_{\mathrm{mRNA}}}{\mathrm{dt}}={\mathrm{k}}_{\mathrm{syn},\mathrm{GRm}} \cdot \left(1-\frac{\mathrm{DR}\left(\mathrm{N}\right)}{{\mathrm{IC}}_{50,\mathrm{GRm}}+\mathrm{DR}\left(\mathrm{N}\right)}\right)-{\mathrm{k}}_{\mathrm{deg},\mathrm{GRm}} \cdot {\mathrm{GR}}_{\mathrm{mRNA}}$$

22 $$\frac{\mathrm{dGR}}{\mathrm{dt}}={\mathrm{k}}_{\mathrm{syn},\mathrm{GR}} \cdot {\mathrm{GR}}_{\mathrm{mRNA}}+{\mathrm{r}}_{\mathrm{f}} \cdot {\mathrm{k}}_{\mathrm{re}} \cdot \mathrm{DR}\left(\mathrm{N}\right)-{\mathrm{k}}_{\mathrm{on}} \cdot \left(\mathrm{CORT}\right) \cdot \mathrm{GR}-{\mathrm{k}}_{\mathrm{deg},\mathrm{GR}} \cdot \mathrm{GR}$$

23 $$\frac{\mathrm{dDR}}{\mathrm{dt}}={\mathrm{k}}_{\mathrm{on}} \cdot \left(\mathrm{CORT}\right) \cdot \mathrm{GR}-{\mathrm{k}}_{\mathrm{T}} \cdot \mathrm{DR}$$

24 $$\frac{\mathrm{dDR}(\mathrm{N})}{\mathrm{dt}}={\mathrm{k}}_{\mathrm{T}} \cdot \mathrm{DR}-{\mathrm{r}}_{\mathrm{f}} \cdot {\mathrm{k}}_{\mathrm{re}} \cdot \mathrm{DR}\left(\mathrm{N}\right)$$

## Data Availability

Simulations and analysis were performed in the MATLAB_R2020b (The MathWorks, http://www.mathworks. com). Additional data and materials generated in this work is available in a public GitHub repository at https://github.com/IPAndroulakis/Light-SCN-HPA.
